# Kisspeptin signaling in astrocytes modulates the reproductive axis

**DOI:** 10.1172/JCI172908

**Published:** 2024-06-11

**Authors:** Encarnacion Torres, Giuliana Pellegrino, Melissa Granados-Rodríguez, Antonio C. Fuentes-Fayos, Inmaculada Velasco, Adrian Coutteau-Robles, Amandine Legrand, Marya Shanabrough, Cecilia Perdices-Lopez, Silvia Leon, Shel H. Yeo, Stephen M. Manchishi, Maria J. Sánchez-Tapia, Victor M. Navarro, Rafael Pineda, Juan Roa, Frederick Naftolin, Jesús Argente, Raúl M. Luque, Julie A. Chowen, Tamas L. Horvath, Vincent Prevot, Ariane Sharif, William H. Colledge, Manuel Tena-Sempere, Antonio Romero-Ruiz

**Affiliations:** 1 Instituto Maimónides de Investigación Biomédica de Córdoba (IMIBIC), Córdoba, Spain.; 2 Department of Cell Biology, Physiology and Immunology, University of Córdoba, Córdoba, Spain.; 3 Hospital Universitario Reina Sofía, Córdoba, Spain.; 4 University of Lille, Inserm, CHU Lille, Laboratory of Development and Plasticity of the Neuroendocrine Brain, Lille Neurosciences & Cognition, UMR-S1172, Lille, France.; 5 Program in Integrative Cell Signaling and Neurobiology of Metabolism, Department of Comparative Medicine, Yale University School of Medicine, New Haven, Connecticut, USA.; 6 Reproductive Physiology Group, Physiology, Development and Neuroscience, University of Cambridge, Cambridge, United Kingdom.; 7 Division of Endocrinology, Diabetes, and Hypertension, Brigham and Women’s Hospital, Harvard Medical School, Boston,Massachusetts, USA.; 8 Centro Fecundacion In Vitro Angela Palumbo, La Laguna, Spain.; 9 CIBER Fisiopatología de la Obesidad y Nutrición, Instituto de Salud Carlos III, Madrid, Spain.; 10 Department of Endocrinology, Hospital Infantil Universitario Niño Jesús, Instituto de Investigación La Princesa, and IMDEA-Food Institute, CEI-UAM+CSIC, Madrid, Spain.; 11 Department of Pediatrics, Universidad Autónoma de Madrid, Madrid, Spain.

**Keywords:** Endocrinology, Reproductive biology, Fertility, Neuroendocrine regulation, Reproductive biochemistry

## Abstract

Reproduction is safeguarded by multiple, often cooperative, regulatory networks. Kisspeptin signaling, via KISS1R, plays a fundamental role in reproductive control, primarily by regulation of hypothalamic GnRH neurons. We disclose herein a pathway for direct kisspeptin actions in astrocytes that contributes to central reproductive modulation. Protein-protein interaction and ontology analyses of hypothalamic proteomic profiles after kisspeptin stimulation revealed that glial/astrocyte markers are regulated by kisspeptin in mice. This glial-kisspeptin pathway was validated by the demonstrated expression of *Kiss1r* in mouse astrocytes in vivo and astrocyte cultures from humans, rats, and mice, where kisspeptin activated canonical intracellular signaling-pathways. Cellular coexpression of *Kiss1r* with the astrocyte markers GFAP and S100-β occurred in different brain regions, with higher percentage in Kiss1- and GnRH-enriched areas. Conditional ablation of *Kiss1r* in GFAP-positive cells in the G-KiR-KO mouse altered gene expression of key factors in PGE_2_ synthesis in astrocytes and perturbed astrocyte-GnRH neuronal appositions, as well as LH responses to kisspeptin and LH pulsatility, as surrogate marker of GnRH secretion. G-KiR-KO mice also displayed changes in reproductive responses to metabolic stress induced by high-fat diet, affecting female pubertal onset, estrous cyclicity, and LH-secretory profiles. Our data unveil a nonneuronal pathway for kisspeptin actions in astrocytes, which cooperates in fine-tuning the reproductive axis and its responses to metabolic stress.

## Abstract 2

A brain non-neuronal pathway for direct kisspeptin actions in astrocytes contributes to reproductive function and its modulation by metabolic status.

## Graphical abstract

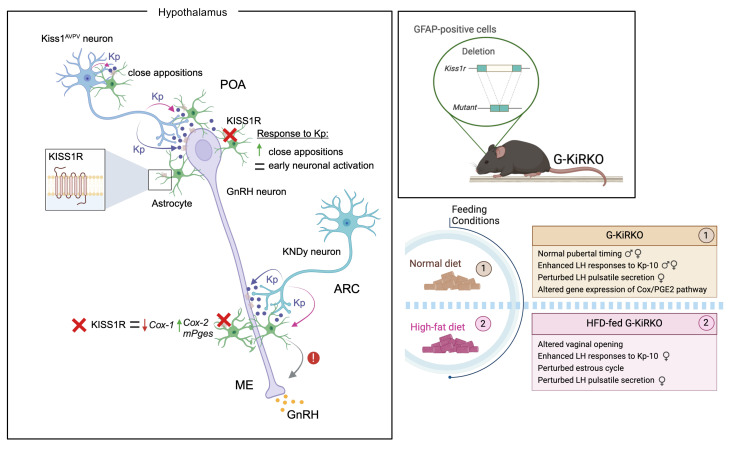

## Introduction

Reproduction, indispensable for continuation of species, is regulated by sophisticated mechanisms, which integrate central and peripheral inputs, acting at different levels of the hypothalamic-pituitary-gonadal (HPG) axis. Reproductive capacity absolutely relies on the pulsatile secretion of gonadotropin-releasing hormone (GnRH), the hypothalamic neuropeptide that drives the secretory pulses of pituitary gonadotropins, luteinizing hormone (LH), and follicle-stimulating hormone (FSH), which, in turn, govern gonadal function (1). GnRH neurosecretion takes place in 2 main patterns: the surge and pulse modes (2). The surge mode occurs exclusively in females and is key for the induction of the preovulatory peak of LH that triggers ovulation. The pulse mode is negatively regulated by sex steroids in both sexes and dictates proper gonadotropin secretory profiles to drive gametogenesis and steroidogenesis.

The central position of GnRH neurons in reproductive control makes them the target of different regulatory pathways. Among these, kisspeptins, encoded by the *Kiss1* gene and acting via the G-protein coupled receptor, KISS1R (aka GPR54), have been recognized as key elicitors of GnRH secretion and essential players in the central regulation of puberty, gonadotropin secretion, and fertility (3). Kisspeptins act primarily on GnRH neurons, which express *Kiss1r* and are potently activated by kisspeptins (1, 3). Functional genomic studies documented that kisspeptin actions on GnRH neurons suffice for attainment of reproductive capacity (4), while elimination of *Kiss1r* selectively from GnRH cells caused central hypogonadism (4, 5). However, *Kiss1r* expression has been found in multiple brain areas not harboring GnRH neurons and central kisspeptin actions at targets other than GnRH neurons are needed for modulation of the reproductive axis (6). However, the nature and physiological relevance of such non-GnRH targets of kisspeptins are ill defined, and the downstream effectors of kisspeptin actions remain largely unknown. No evidence for nonneuronal targets of kisspeptins at central levels has been presented to date.

Two main populations of Kiss1 neurons have been found in the hypothalamus, one in the arcuate nucleus (ARC) and the other in the rostral hypothalamic area, mainly the anteroventral periventricular nucleus (AVPV) in rodents (3). ARC Kiss1 neurons, which are found in both sexes, mediate the negative feedback effect of sex steroids and are a key component of the GnRH pulse generator (7). In contrast, AVPV Kiss1 neurons display a clear sex dimorphism, with predominant presence in females, and are involved in mediating the positive feedback effect of estradiol and the generation of the preovulatory GnRH/LH surge (8). Projections of Kiss1 neurons to multiple hypothalamic and extrahypothalamic areas have been documented (9), while nonsynaptic contacts of Kiss1 neurons with GnRH neurons have been recently documented, suggesting volume transmission (10).

GnRH neurosecretion is also modulated by glial cells, of which astrocytes are the most abundant subtype. Astrocytes are known to play a critical role in the regulation of reproductive function; the bidirectional interaction between astrocytes and GnRH neurons and their adhesiveness are essential for proper reproductive control (11–13). Astrocytes are abundantly located in the vicinity of GnRH neurons (14) and ensheath them, with several adhesion factors being expressed in both astrocytes and GnRH neurons to permit homophilic interactions (12). Plastic changes in astrocyte morphology and their contacts with GnRH neurons have been demonstrated in different states of the reproductive axis (15). Moreover, astrocytes respond to key reproductive regulators, such as gonadal steroids (16), and produce different signals, including growth factors (e.g., IGF-1, TGF-β, and EGF family members), neurotransmitters (e.g., glutamate) and prostaglandins (e.g., PGE_2_) (11, 12, 17), that modulate GnRH neurosecretory activity. Astrocytes are also sensitive to metabolic cues, e.g., leptin and ghrelin (18, 19), known to influence reproductive function, and insulin was recently shown to act in astrocytes to funnel at least part of its modulatory actions on puberty onset and gonadal function in mice (20). In the context of our search for novel brain targets of kisspeptins, we report herein the characterization of a pathway involving kisspeptin signaling in astrocytes, a nonneuronal target in the brain, initially identified by proteomic analyses and defined further by expression and functional genomic studies.

## Results

### Proteomic identification of novel kisspeptin targets in the hypothalamus.

We used *Kiss1*-null mice to identify hypothalamic targets modulated by kisspeptins, by applying quantitative proteomics following a bolus of kisspeptin-10 (Kp-10). Since Kiss1-KO mice are devoid of endogenous levels of kisspeptin (21), we hypothesized this would increase the capacity to detect changes in expression of kisspeptin-responsive proteins after stimulation with an effective but submaximal dose of Kp-10 (50 pmol), selected to avoid supraphysiological stimulation. To exclude detection of rapid posttranslational changes (e.g., in intracellular signaling cascades), hypothalamic tissue was obtained 60 min after i.c.v. injection of Kp-10, a time-point when a significant elevation of serum LH levels was detected (4.41 ± 0.25 ng/mL versus 1.0 ± 0.30 ng/mL in vehicle-treated animals; *P*=0.0012), confirming the efficacy of the dose and time selected to activate the gonadotropic axis (22).

**Figure 1:**
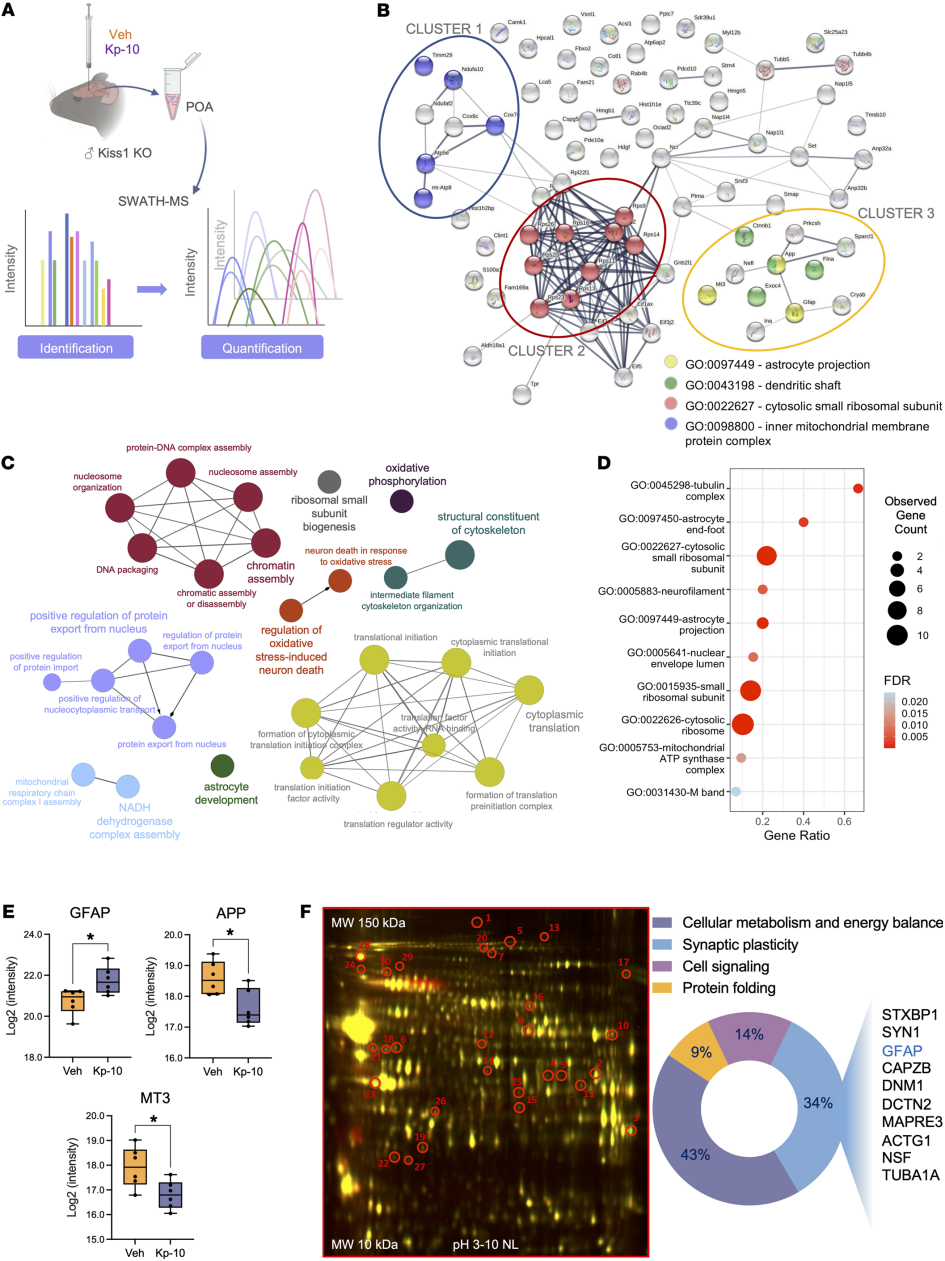
Identification of kisspeptin targets in POA by proteomic analysis. (**A**) Scheme of experimental design to identify new targets of kisspeptin actions in adult Kiss1-KO male mice (*n* = 6 per group), using SWATH-MS method. (**B**) High throughput data (77 differentially expressed proteins found) were analyzed via STRING to build functional protein association networks (the 3 main clusters circled correspond to GO terms). Analyses were also implemented by (**C**) enrichment analyses in GO categories such as biological process visualized by Cytoscape platform and (**D**) cellular components using *ggplot2* R package (cut-off r > 0.800). (**E**) box-plots represent the intensity of GFAP, APP and MT3 proteins from SWATH-MS raw data. Data are the mean ± SEM. Statistical significance was determined by Student’s *t* test: *P < 0.05 versus corresponding values in adult Kiss1-KO mice treated with vehicle (Veh). (**F**) 2D-DIGE map (left panel) and pie chart (right panel) presenting the GO of enriched proteins from an independent validation of Kp-10 effects on Kiss1 KO mice. Red circles highlight differential protein expression in POA from Kiss1-KO mice after Kp-10 injection (*n* = 3) versus vehicle-treated mice (*n* = 3). Spots were identified by MALDI-MS/MS.

Nano–high-performance liquid chromatography (nano-HPLC), equipped with sequential window acquisition of all theoretical mass spectra (SWATH) acquisition for label-free quantitative proteomics, identified a set of differentially expressed proteins in the hypothalamic preoptic area (POA), following i.c.v. injection of Kp-10 (Figure 1A). These proteins were categorized/displayed in protein-protein interaction (PPI) networks, biological processes defined by gene ontology (GO), and enrichment analyses based on cellular component GO terms (TOP10). The STRING database identified PPI networks of 77 differentially expressed proteins, with 3 main clusters being found. CLUSTER-1 included components of the mitochondrial respiratory chain. The most robust association, CLUSTER-2, was centered around ribosomal proteins (RPs), while an independent network, CLUSTER-3, was organized around glial fibrillary acidic protein, GFAP, a major component of cytoskeleton and putative marker of astrocytes (23) (Figure 1B).

GO-enrichment analyses identified different biological pathways putatively modulated by kisspeptin in the hypothalamic POA, including, among others, cytoplasmic translation, structural constituents of the cytoskeleton, oxidative phosphorylation, regulation of oxidative stress-induced neuron death and, notably, astrocyte development (Figure 1C). Of the top 10 variables identified by an enrichment analysis using GO terms, astrocyte end-foot and astrocyte projections, identified in CLUSTER-3, were classified as 2 of the most significant categories based on their adjusted *P* values and gene ratios, suggesting a potential modulation of astrocyte-molecular processes by kisspeptin (Figure 1D).

We further analyzed the raw SWATH-MS data in order to provide individual validation of the changes in GFAP, Amyloid Precursor Protein (APP) and Metallothionein 3 (MT3) levels in the POA after kisspeptin stimulation; APP and MT3 were also visualized in CLUSTER-3 of the PPI network. Our analyses documented that kisspeptin upregulated GFAP levels in the POA, whereas the expression levels of APP and MT3 were significantly decreased following Kp-10 injection to Kiss1-KO mice (Figure 1E).

In a parallel confirmatory approach, 2-dimensional difference gel electrophoresis (2D-DIGE) was applied to an independent set of POA samples from Kiss1-KO mice i.c.v. injected with Kp-10. DIGE analyses detected 30 differentially expressed proteins, identified by MS. Ontology analysis revealed factors involved in cellular metabolism and energy balance, cell signaling, protein folding, and synaptic plasticity. Of note, approximately 34% of these differentially expressed proteins were allocated to the category of synaptic plasticity and included GFAP as an altered protein in response to kisspeptin (Figure 1F). These DIGE results confirmed our data from SWATH-based quantitative proteomics, suggesting kisspeptin regulation of key astrocyte markers.

### Characterization of a kisspeptin pathway in astrocytes.

**Figure 2:**
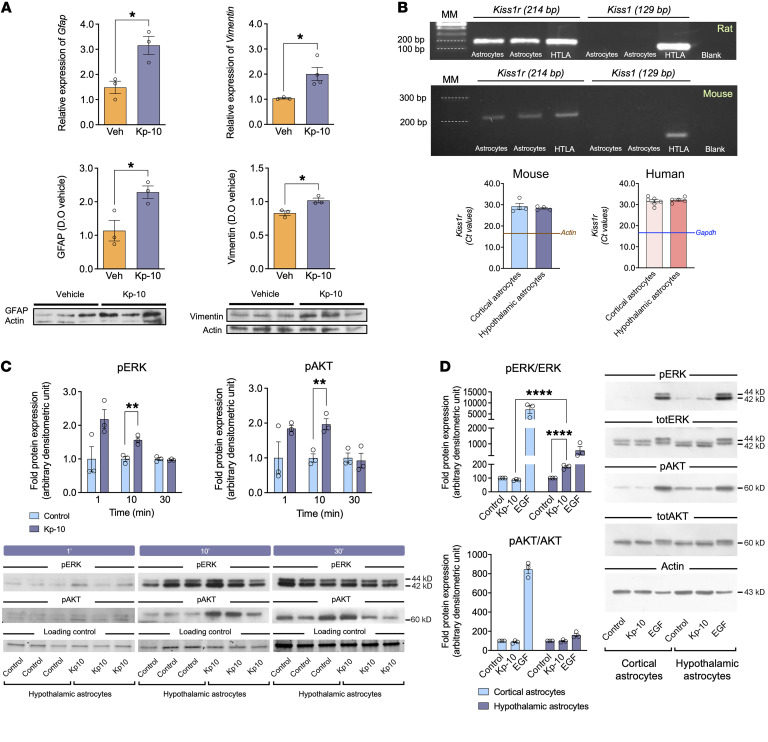
Evidence for kisspeptin signaling in astrocytes. (**A**) Expression analysis of glial markers *Gfap/*GFAP and *Vimentin*/Vimentin at mRNA and protein levels in POA of adult Kiss1-KO male mice after i.c.v. Kp-10 stimulation (*n* = 3–4) versus vehicle (*n* = 3). Data are the mean ± SEM. Statistical significance was determined by Student’s *t* test: *P < 0.05 versus KO mice treated with vehicle. (**B**) Representative gels illustrating the expression of *Kiss1r*, but not *Kiss1* mRNA in 2 pools of primary astrocyte cultures from neonatal rat (upper gel) and mouse (lower gel) hypothalamus are presented. Hypothalamic (HTLA) tissue was used as positive control. MM, molecular markers. Real-time PCR of *Kiss1r* mRNA in primary mouse and human astrocyte cultures (*n* = 4 for mouse; *n* = 6 cortical and 5 hypothalamic human cultures) is also shown; values correspond to Ct data. The blue line represents the mean Ct value of the housekeeping gene. In (**C**), Western blots of phosphorylated ERK (pERK) and AKT (pAKT) in primary rat hypothalamic astrocytes are shown. Bar graphs show the effect of Kp-10 treatment (10^–8^ M; *n* = 3) at 1, 10, and 30 minutes (upper panel); representative blots are shown in the lower panel. Astrocyte cultures treated with vehicle (*n* = 3) were used as a negative control. Data are the mean ± SEM. Statistical significance was determined by Student’s *t* test: **P < 0.01 versus astrocytes treated with vehicle. (**D**) Western blots of pERK, total ERK (totERK), pAKT, total AKT (totAKT), and actin, in primary mouse cerebrocortical and hypothalamic astrocytes treated with Kp-10 (*n* = 3) or Epidermal Growth Factor (EGF, 50 ng/mL; *n* = 3), used as a positive control. Vehicle-treated astrocytes (*n* = 3) were used as a negative controls. Data are the mean ± SEM. Statistical significance was determined by 2-way ANOVA followed by Bonferroni’s post hoc test: ****P < 0.0001, astrocytes treated with Kp-10 versus vehicle; or cortical versus hypothalamic astrocytes.

To confirm the putative regulatory actions of kisspeptin in astrocytes, the expression of GFAP and vimentin, which is also produced in developing and activated astrocytes, was independently evaluated at the mRNA and protein levels in the POA of Kiss1-KO mice following i.c.v. injection of Kp-10. These analyses confirmed the proteomic data since expression of both putative astrocyte markers was increased in POA after central kisspeptin stimulation (Figure 2A). *Kiss1r* mRNA expression was assessed also in primary astrocyte cultures from WT neonatal rats and mice as well as humans. Expression of *Kiss1r* was demonstrated in astrocytes of both rodent species and humans, with comparable cycle threshold (Ct) values in real-time PCR analyses between humans and mice. In contrast, *Kiss1* expression was not detected in astrocyte cultures from rats or mice (Figure 2B).

Using primary cultures from neonatal rodents, we interrogated whether key elements of the canonical intracellular signaling pathways are activated by kisspeptin in astrocytes. Kp-10 treatment induced the phosphorylation of ERK/MAP kinase, a pivotal component of the kisspeptin signaling pathway in target cells (24) in rat hypothalamic astrocytes, with peak levels at 10 minutes after stimulation (Figure 2C). In addition, an increase in the level of phosphorylation of AKT was observed at 10 minutes after kisspeptin stimulation. Kp-10 treatment also stimulated the phosphorylation of ERK/MAP kinase in primary cultures of mouse hypothalamic astrocytes but not in cortical astrocytes. In mice, however, Kp-10 did not stimulate the phosphorylation of AKT in either hypothalamic or cortical astrocytes (Figure 2D).

**Figure 3:**
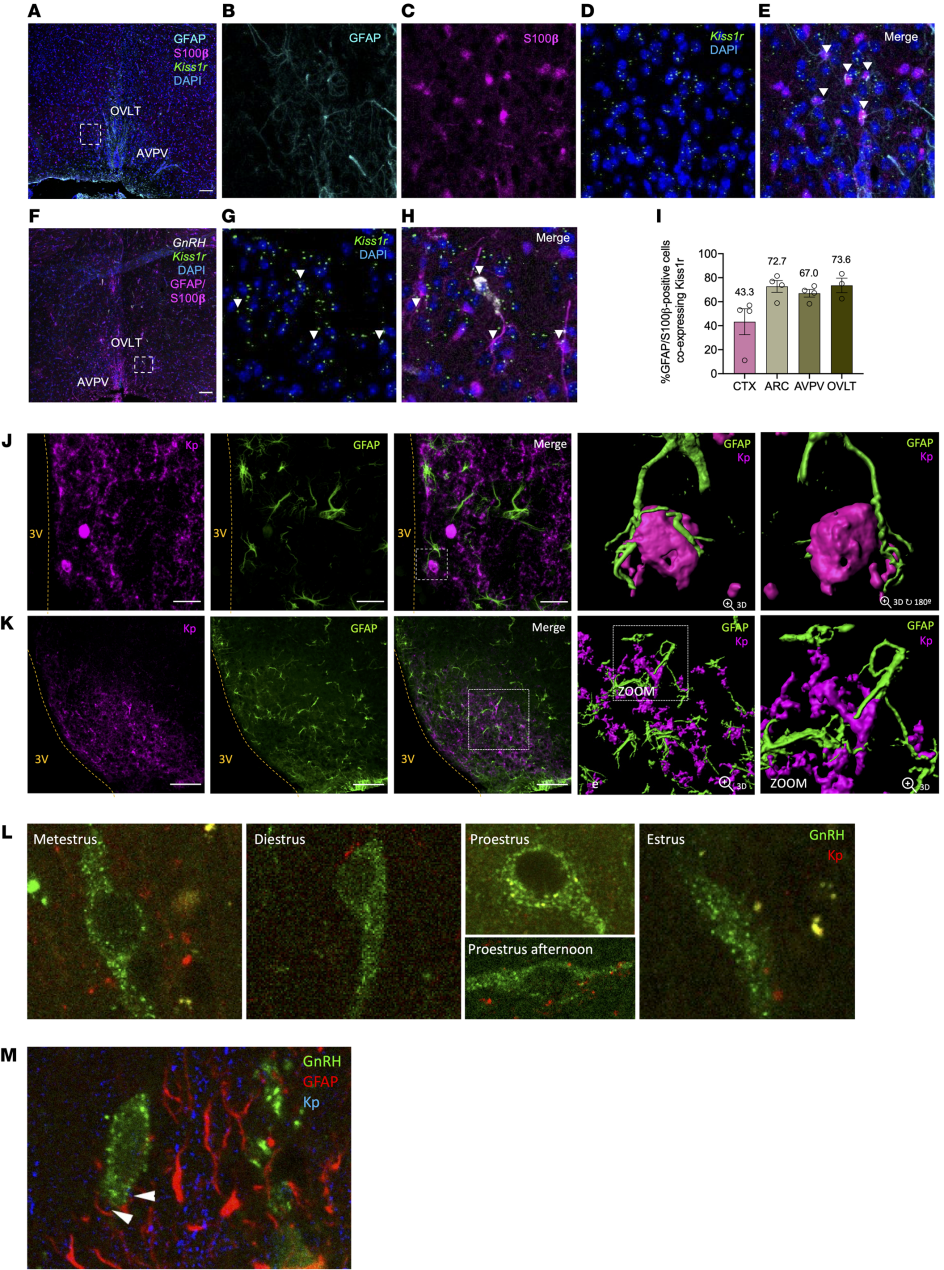
Coexpression of Kiss1r in astrocytes and evidence for direct astrocyte-Kiss1 neuron interplay. (**A**–**H**) Dual RNAscope ISH combined with IHC in brain sections from diestrous female mice (*n* = 4). (**A**) Representative image showing *Kiss1r* (green) mRNA and GFAP (cyan) and S100-β (magenta) in the preoptic region. The magnified area (from dotted square in **A**) shows individual signals (**B**–**D**), while merge image documents coexpression of *Kiss1r* in GFAP/S100-β–positive cells (arrowheads; **E**). (**F**) Representative image of *Kiss1r* (green) and *GnRH* (white) mRNA expression and combined detection of astrocyte markers GFAP and S100-β proteins (magenta) in POA, including OVLT and AVPV. The magnified area (from dotted square in **F**) shows *Kiss1r* expression and neuronal nuclear labelling with DAPI (blue; **G**), while coexpression (arrowheads) of *Kiss1r* with *GnRH* and *Kiss1r* with astrocyte markers is shown in **H**. (**I**) Percentage of GFAP/S100-β–positive cells coexpressing *Kiss1r* mRNA in key hypothalamic areas, including ARC and AVPV, OVLT, and cortex (CTX). Scale bars (**A**–**F**): 100 μm. Data are the mean ± SEM. (**J** and **K**) Anatomical relationships between Kp-immunoreactive neurons and GFAP-positive astrocytes from diestrous female mice. Individual and merged images of Kp (magenta) and GFAP (green) are presented from AVPV (**J**) and ARC (**K**); 3D reconstructions of GFAP-immunoreactive astrocytes enwrapping cell bodies of Kp cells in AVPV are also shown (**J**); close appositions between GFAP-immunoreactive astrocytes and Kp fibers are detected in ARC at high magnification (**K**). Scale bars: 50 μm (**J**); 100 μm (**K**). (**L**) Representative images of GnRH neurons in close apposition with Kp fibers are shown at the stages of the ovarian cycle (10:00 a.m.); an additional image at proestrus afternoon is shown. Merge images of GnRH neurons (green) and Kp fibers (red) in the medial septal nucleus are presented. Images were taken with total 80× magnification. (**M**) Higher magnification (×2) of a representative image, with triple labeling of GnRH-neurons (green), Kp-fibers (blue) and GFAP-positive cells (red), in the hypothalamic medial septal nucleus.

We also assessed whether astrocyte markers GFAP and S100-β are coexpressed with *Kiss1r* in vivo in various brain areas of control female mice at diestrus. Double labelling analyses, using RNAscope in situ hybridization (ISH) to detect *Kiss1r* and *GnRH* mRNA and IHC to detect GFAP- and S100β-positive cells, conclusively showed not only that GnRH neurons coexpress *Kiss1r*, in line with previous reports (25), but also that *Kiss1r* expression is detectable in GFAP/S100-β–expressing cells in different hypothalamic areas involved in the control of the reproductive axis, as the organum vasculosum lamina terminalis (OVLT), AVPV and ARC, as well as the cortex, used as nonneuroendocrine reference control. For representative examples of individual labeling, and *Kiss1r*/GFAP/S100β or *Kiss1r*/*GnRH*–positive cells in the OVLT, see Figure 3, A–H. Quantitative analyses documented an enrichment in the percentage of colocalization of *Kiss1r* and GFAP/S100-β in OVLT (73%) and AVPV (67%), areas in which GnRH and Kiss1 neuronal populations are found, as well as in the ARC (72%), where terminals of GnRH neurons project into the median eminence and the other prominent Kiss1 neuronal population is found. In the cortex, although there was detectable colocalization between *Kiss1r* and GFAP/S100-β, the percentage of double-positive cells was lower than in the former brain areas (Figure 3I).

In addition, we explored the existence of intimate appositions between astrocytes and Kiss1 neurons as anatomical substrate for kisspeptin actions on *Kiss1r*-expressing astrocytes in vivo. We applied IHC detection of GFAP and kisspeptin in control female mice with particular focus on the AVPV and ARC. Confocal images and 3D reconstructions documented clear close appositions between astrocyte processes (denoted by GFAP-immunoreactivity) and Kiss1 neurons at the level of cell bodies and fibers in the AVPV and ARC, respectively (Figure 3, J and K). These contacts were consistently observed across the different AVPV and ARC sections and individuals studied. We also evaluated changes in the number of contacts between kisspeptin fibers and GnRH neurons across the ovarian cycle and the potential interaction with astrocyte processes. While in metestrus and diestrus, approximately 40% of GnRH neurons were contacted by kisspeptin fibers; the number of appositions drop dramatically to less-than 10% in the morning (10:00 am) of proestrus, i.e., before the initiation of the preovulatory surge, but raised thereafter through the early afternoon of proestrus (55%) until the morning of estrus, when they reached values of over 70% of GnRH neurons receiving kisspeptin contacts (Figure 3L). Notably, kisspeptin fibers, when in contact with GnRH neurons, were in close proximity to GFAP-labeled processes (Figure 3M).

**Figure 4:**
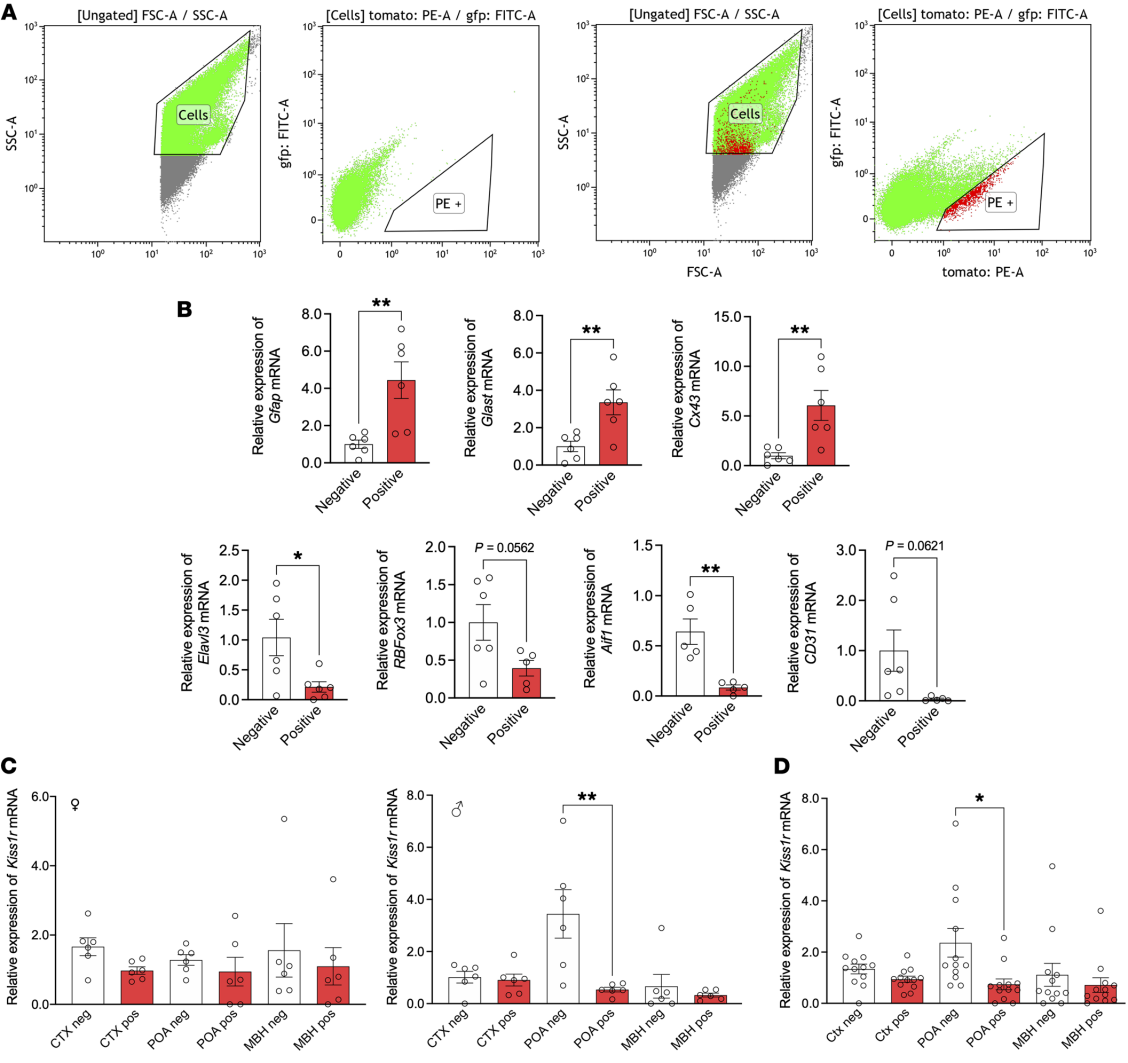
Kiss1r expression in astrocytes from adult mice isolated by FACS. (**A**) Gating strategy for astrocyte isolation by FACS. The 2 plots in the left represent cells incubated with the control isotype; the 2 plots in the right represent cells incubated with the anti-ACSA-2-PE antibody. (**B**) Real-time PCR of astroglial [*Gfap, Glast, Connexin-43* (Cx43)], neuronal (*Elavl3, RBFox3*), microglial (*Aif1*), and endothelial (*CD31*) genes in FACS-sorted positive and negative fractions of the MBH of female mice, used for validation purposes. (**C**) Real-time PCR analysis of *Kiss1r* in FACS-sorted positive and negative fractions obtained from 3 brain areas (POA, MBH, and cortex [CTX]) of adult male and female (diestrus) mice. Expression data segregated by sex (females, left panel; males, right panel) are presented. Sex-aggregated data, divided per brain region, are displayed in **D**. *n* = 6 animals per sex. Values in the positive fraction are expressed relative to negative fraction values, set at 1. Data are the mean ± SEM. Statistical significance was determined by Student’s *t* test in **B**: *P < 0.05; **P < 0.01, versus corresponding negative fraction; and by 2-way ANOVA followed by Bonferroni’s post hoc test for regional and sex analyses in **C** and **D**: *P < 0.05; **P < 0.01 versus negative fraction. Note that of the 36 positive fractions (3 regions × 2 sexes × 6 animals per group) tested**,** *Kiss1r* expression was readily detectable in 33, with a mean Ct value of 21.9.

Astrocytic expression of *Kiss1r* in vivo was documented also by qPCR analyses of astrocytes isolated from control male and female mice using fluorescence-activated cell sorting (FACS). Sorting procedures were optimized and validated by expression analyses in the mediobasal hypothalamus (MBH) of female mice, which documented substantial enrichment of astrocyte-abundantly expressed genes, such as *Gfap*, *Glast,* and *Cx43*, in the astrocyte-positive fraction, whereas neuronal-expressed (*Elavl3/Huc*, *RBFox3/NeuN*), microglial-expressed (*Aif1/Iba1*), and endothelial-expressed (*CD31*) genes were enriched in the negative fraction (Figure 4, A and B). Expression analyses of *Kiss1r* in astrocytes obtained from the POA, MBH, and cortex of male and female mice revealed unambiguous expression in the astrocyte-positive fraction in all areas analyzed, with grossly similar profiles in both sexes (Figure 4C). In fact, of the 36 positive fractions (3 regions × 2 sexes × 6 animals per group) tested, *Kiss1r* expression was detectable in 33, with a mean Ct value of 21.9. Aggregated expression analysis per region documented higher levels in the FACS-negative versus positive fraction only in the POA, in line with the abundant expression of *Kiss1r* in GnRH neurons (3), with *Kiss1r* expression being detectable in the 3 areas analyzed (Figure 4D).

### Analysis of kisspeptin signaling in astrocytes in vivo: Studies in the G-KiR-KO mouse.

To interrogate the physiological relevance of direct kisspeptin actions in astrocytes, we generated a mouse line with conditional ablation of *Kiss1r* in GFAP-expressing cells by crossing a well-validated Gfap-Cre mouse line (26) with a *Kiss1r* ^lox/lox^ mouse line, previously used in our group for kisspeptin receptor ablation in vivo (27, 28). This mouse line was named G-KiR-KO, for *GFAP-specific Kiss1 Receptor KO* (Supplemental Figure 1A; supplemental material available online with this article; https://doi.org/10.1172/JCI172908DS1). To validate this line, PCR was used to detect the recombination event at the loxP sites of *Kiss1r* gene, denoting effective Cre activity and gene inactivation. Effective recombination took place in the brain of G-KiR-KO mice, abundantly in the POA and, to a lesser extent, in the MBH (Supplemental Figure 1B). Very low-to-negligible recombination was detected in the lung or white and brown adipose tissues, as reference peripheral tissue controls, while recombination was also found in the testis, where Gfap expression has been documented in Leydig cells (29).

In line with effective hypothalamic recombination, *Kiss1r* mRNA levels in astrocyte cultures from G-KiR-KO mice were low to negligible, in contrast to control astrocytes and hypothalamic tissue from control mice (Supplemental Figure 1B). Functional studies in primary cultures of astrocytes from the hypothalamus of control and G-KiR-KO mice evidenced a significant increase (over 35%) in phospho-ERK levels 10 minutes after Kp-10 treatment in astrocytes of control mice, while no significant changes in ERK phosphorylation were observed in astrocytes from G-KiR-KO mice after kisspeptin challenge. Detectable expression of GFAP, but not the neuronal marker, NeuN, was found in our astrocyte primary cultures, denoting glial lineage and absence of neuronal contamination. No significant change in GFAP protein content was found in cultures from control or G-KiR-KO mice after short-term (10 minute) challenge with Kp-10 (Supplemental Figure 1C), seemingly due to the small window of stimulation.

Further evidence for targeted Cre activity in astrocytes in vivo was obtained using a reporter mouse line, which was generated by crossing the Gfap-Cre mouse with a reporter line in which YFP (yellow fluorescent protein) is expressed upon Cre-mediated recombination. Using GFAP and S100-β as astrocyte makers to detect not only processes but also the cytosolic shape of astrocytes, we found that about 92%, 89%, and 95% of GFAP/S100-β–positive cells coexpressed YFP (denoted by GFP-immunostaining) in the ARC, AVPV, and OVLT areas, respectively (Supplemental Figure 2, A and C). This strong astrocyte-predominant effective recombination was also supported by the fact only 25%–30% of nonastrocytic, NeuN-positive cells in the above areas displayed GFP immunoreactivity (Supplemental Figure 2, B and D).

**Figure 5:**
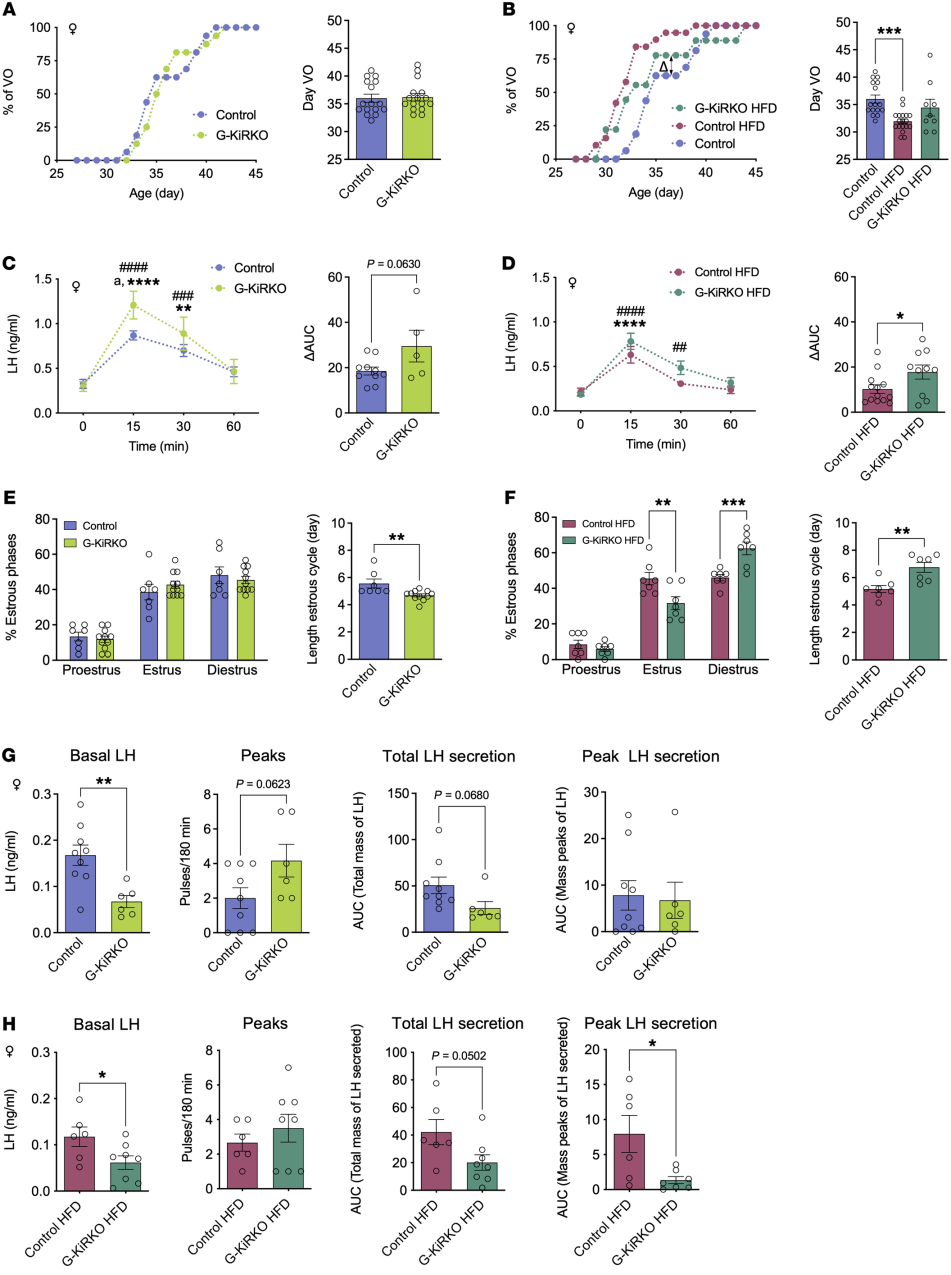
Characterization of reproductive phenotype of G-KiR-KO female mice. In upper panels*,* accumulated percentage of female mice displaying vaginal opening (VO; as pubertal marker) postweaning, under normal diet (**A**) or HFD (**B**); mean ages of VO are presented as histograms. Group sizes: control (*n* = 16); G-KiR-KO (*n* = 16); control-HFD (*n* = 18); and G-KiR-KO-HFD (*n* = 9). Statistical significance for mean VO was assessed by Student’s *t* test (**A**) or 1-way ANOVA followed by Bonferroni’s test (**B**) ***P < 0.001 versus control mice. LH secretory responses, as 60 minute profile after Kp-10 injection (50 pmol), are shown for adult control and G-KiR-KO female mice fed normal diet (**C**) or HFD (**D**); net increment of integral (AUC) LH secretion over 60 minute period after Kp-10 is also presented. Group sizes: control (*n* = 10); G-KiR-KO (*n* = 5); control-HFD (*n* = 13); and GKiR-KO-HFD (*n* = 10). Statistical significance was determined by Student’s *t* test: *P < 0.05 versus control mice (AUC); and 2-way ANOVA followed by Bonferroni’s test for time-course analyses: **^/##^*P* < 0.01; ^###^*P* < 0.001 and ****^/####^*P* < 0.0001 versus corresponding basal (time-0) values; and ^a^ *P* < 0.05 G-KiR-KO versus control mice. (**E** and **F**) Graphs showing the percentage distribution of estrous cycle phases in control and G-KiR-KO mice for normal diet (**E**) and HFD (**F**); control (*n* = 7); G-KiR-KO (*n* = 12); control-HFD (*n* = 7) and G-KiR-KO-HFD (*n* = 7). Mean duration of estrous cycle is displayed also. Statistical significance was determined by Student’s *t* test (**E**) **P < 0.01 versus control mice with normal diet; and by 2-way ANOVA followed by Bonferroni’s test (**F**) **P < 0.01; ***P < 0.001 versus control mice fed with HFD. (**G**) LH pulsatility parameters in G-KiR-KO mice fed control diet are shown; control (*n* = 9), G-KiR-KO (*n* = 6). Bar graphs showing basal LH, numbers of LH pulses (peaks), net increment (AUC) LH secretion, and peak LH secretion over 3 hour sampling are presented. (**H**) Similar parameters are shown for G-KiR-KO mice under a HFD; control (*n* = 6), G-KiR-KO (*n* = 8). Student’s *t* test: *P < 0.05; **P < 0.01 versus. control mice.

G-KiR-KO mice were assessed for somatic and pubertal maturation, both under normal (chow) diet and after metabolic challenge with 58% high-fat diet (HFD) from weaning. No differences in body weight (BW) gain were detected between genotypes, irrespective of the feeding regimen, either in females (Supplemental Figure 3, A and B) or males (Supplemental Figure 4, A and B). G-KiR-KO female mice under chow diet displayed conserved ages of puberty onset, denoted by vaginal opening (VO; Figure 5A), and first estrus (FE; Supplemental Figure 3C). In contrast, while control females fed a HFD showed a marked advancement of the mean age of VO, this effect was blunted in G-KiR-KO female mice under HFD, whose mean age of VO was similar to that of control mice fed chow diet (Figure 5B). Yet, no clear differences were detected in the age of FE between control and G-KiR-KO mice fed a HFD (Supplemental Figure 3D). In adulthood, i.c.v. stimulation with a submaximal dose of Kp-10 (50 pmol) evoked significant LH secretory responses in female control and G-KiR-KO mice on a chow diet, in line with previous studies (22). Yet, the magnitude of Kp-induced LH secretion was significantly higher at peak levels, 15 minutes after Kp-10 injection, in G-KiR-KO females (Figure 5C). Female G-KiR-KO mice on a HFD also displayed higher LH secretory responses to Kp-10 simulation (Figure 5D). In addition, HFD exposure induced estrous cycle irregularities in female G-KiR-KO mice, with longer cycle length and a higher number of days in diestrus and reduced number of days in estrus. These alterations were not detectable in lean female G-KiR-KO mice, as they did not present overt perturbations in individual phases of ovarian cyclicity (Figure 5, E and F), except for a moderate shortening in the total length of the cycle. Despite the lack of major cycle irregularities, LH secretory patterns in female G-KiR-KO mice displayed notable alterations, with a significant lowering of basal LH levels and a trend toward a higher number of LH secretory pulses (peaks) in G-KiR-KO animals fed a chow diet. Due to variability within the control group, total LH secretion and LH secretory mass per pulse were not significantly decreased, despite a strong trend for decline in total LH secretion (Figure 5G). No change in the magnitude of estrogen-primed LH surges, nor overt alterations in fecundity indices, were detected between control and G-KiR-KO female mice (Supplemental Figure 3, E and F). HFD feeding to female G-KiR-KO mice worsened LH secretory profiles, as denoted by significantly lower basal LH levels and LH secretory mass per pulse, as well as a strong trend to decline in total LH secretion (Figure 5H). For representative individual LH secretory profiles of female G-KiR-KO mice, fed chow or HFD, see Supplemental Figure 5, A and B.

Reproductive phenotypic markers were less affected in GKiR-KO males, which did not show changes in the age of puberty onset, denoted by balano-preputial separation (BPS), either under chow diet or HFD (Supplemental Figure 4, C and D). In adulthood, adult male control and G-KiR-KO mice showed robust LH responses to 50 pmol i.c.v. Kp-10 stimulation, which were significantly higher at 15 minutes in G-KiR-KO versus controls. Yet, in contrast to females, G-KiR-KO males on HFD had LH responses to Kp-10 similar to those of control mice under a HFD (Supplemental Figure 4E).

The potential impact of congenital deletion of *Kiss1r* in astrocytes on key metabolic parameters was also monitored in adult animals of both sexes. No differences in terms of either BW or body fat and lean mass were detected between genotypes, in either sex or feeding regime (chow versus HFD; Supplemental Figure 6, A and B). Regarding glucose homeostasis, basal glucose levels were similar in control and G-KiR-KO mice of both sexes. However, glucose-tolerance tests (GTT) revealed a subtle improvement of the response to a glucose bolus in G-KiR-KO mice, as denoted by time-course profiles and integral AUC glucose values, over the 120 minute period, which were significantly lower in G-KiR-KO male mice on a chow diet. A similar trend was observed in females, but the reduction in AUC for glucose during the GTT was slightly below the level of statistical significance (Supplemental Figure 7, A and B). In addition, a moderate improvement of glucose tolerance was also noted in G-KiR-KO males fed a HFD, which was not detected in null HFD females. G-KiR-KO mice of both sexes did not display consistent alterations in insulin sensitivity, measured by insulin-tolerance tests (ITT), under normal or HFD conditions (Supplemental Figure 7, C and D).

The consequences of specific ablation of *Kiss1r* in astrocytes in terms of their interplay with GnRH neurons were also evaluated. Peripheral administration of an effective dose of Kp-54 was applied, as previously reported (30), and both cFos activation in GnRH neurons and changes in appositions between GFAP-positive and GnRH cells were analyzed. Potent LH responses were found in control and G-KiR-KO female mice at 60 minutes after Kp-54 injection (Supplemental Figure 8). No significant differences were detected in the percentage of GnRH neurons expressing cFos between genotypes, albeit considerable variability was observed (Supplemental Figure 8, A and D). However, the number of close appositions between GFAP-positive cells and GnRH neurons was increased in G-KiR-KO mice, with this difference reaching statistical significance for interactions at the level of the soma (Supplemental Figure 8, B, E, and F), denoting that elimination of kisspeptin signaling in astrocytes may perturb their physical interplay with GnRH neurons.

**Figure 6:**
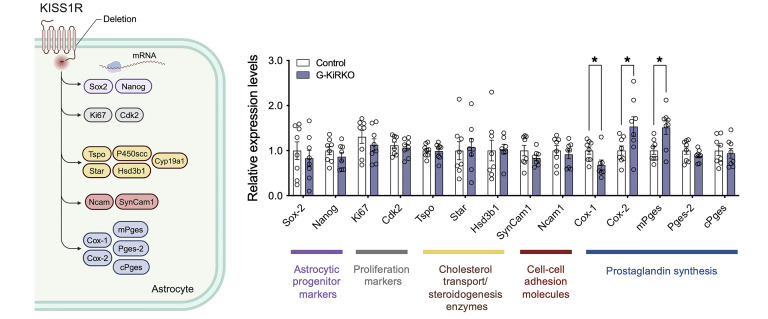
Gene expression profiling in G-KiR-KO astrocyte primary cultures. A comprehensive overview of the set of genes whose expression was analyzed by qPCR in astrocyte cultures of G-KiR-KO mice is shown in the left panel. Gene categories correspond to astrocyte progenitors (purple), astrocyte proliferation (grey), cholesterol transport, and steroidogenesis (yellow-brown), cell-cell adhesion interaction (red), and prostaglandin synthesis (blue). In the right panel, quantitative data from qPCR expression analyses conducted in duplicate in individual astrocyte cultures from control (*n* = 4) and G-KiR-KO (*n* = 4) mice. The expression levels of *Sox-2*, *Nanog*, *Ki67*, *Cdk2*, *Tspo*, *Star*, *Hsd3b1*, *SynCam1*, *Ncam1*, *Cox-1*, *Cox-2*, *mPges*, *Pges-2,* and *cPges* mRNA are shown after normalization using *S11* expression levels. Note that *P450scc* and *P450arom* displayed virtually undetectable expression levels in our cultures, and hence are not presented in the histograms. Data are shown as mean ± SEM. Statistical significance was determined by Student’s *t* test, *P < 0.05 versus corresponding values in control astrocytes.

Finally, qPCR analyses were applied to primary cultures of astrocytes from control and G-KiR-KO mice to assess the expression levels of a set of genes involved in key aspects of astrocyte physiology. These included molecular factors involved in astrocyte differentiation and proliferation (31–34), elements of the steroidogenic pathway (35), adhesion molecules involved in glia-to-GnRH neuron interactions (36), and factors involved in prostaglandin (PG) synthesis (37). No gene expression changes were detected for differentiation, proliferation, steroidogenic, or adhesion factors. In contrast, mRNA levels of some components of the PG synthesis pathway were altered in astrocytes from G-KiR-KO mice. Thus, the expression levels of cyclooxygenase genes, *Cox-1* and *Cox-2*, were oppositely changed, with decreased *Cox-1* expression and increased *Cox-2* mRNA levels in G-K-KO astrocytes. In addition, expression of the gene encoding the inducible microsomal PG E synthase-1 (*mPges*) was significantly increased in astrocytes lacking *Kiss1r*. Gene expression of other constitutive factors of the PG synthetic pathway, as *Pges2* and *cPges*, was not altered in astrocyte cultures from G-KiR-KO mice (Figure 6).

## Discussion

Characterization of the entire set of cellular and molecular pathways underlying kisspeptin actions in the hypothalamus remains incomplete. In our search for protein targets of kisspeptins in the POA, i.e., where most GnRH neurons are located (1), we applied label-free quantitative proteomics to identify individual factors as well as cellular and molecular pathways modulated by kisspeptin. To maximize our discrimination capacity, we used Kiss1-KO mice, devoid of endogenous kisspeptins, as optimal for detection of protein targets up- or downregulated after challenge with exogenous kisspeptin. Admittedly, congenital Kiss1 null mice display hypogonadotropic hypogonadism, which might cause some developmental defects due to lower sex steroid levels. However, importantly for the purposes of our study, they retain proper migration of GnRH neurons into the hypothalamus and kisspeptin responsiveness (21).

PPI analyses on SWATH data allowed identification of proteomic responses to kisspeptin stimulation, as defined by different protein clusters, including elements of the respiratory chain as well as RPs and other factors involved in protein translation, possibly reflecting activation of basic cellular processes needed for kisspeptin effects. Further, our complementary 2D-DIGE proteomic approach revealed different categories regulated by kisspeptin, including proteins involved in cell metabolism, cell signaling, and protein folding in line with the variety of intracellular cascades mediating kisspeptin actions. These kisspeptin-sensitive pathways warrant independent investigation. It must be stressed, however, that our proteomic approach was not directed toward identification of conventional intracellular signaling mediators, e.g., we did not search for rapid phosphoproteomic changes, but aimed to pinpoint major downstream elements of kisspeptin actions amenable for confirmation by physiological studies, assuming that the genetic model and time window of analysis could hamper identification of the whole repertoire of kisspeptin targets.

In this scenario, we were especially attracted by our findings on the putative regulation of astrocytic-related markers by kisspeptin. Indeed, PPI analyses revealed an independent cluster modulated by kisspeptin, centered around GFAP, in which other proteins, such as MT3 and APP, were also identified. MT3 is a zinc-biding metallothionein that contributes to actin polymerization in astrocytes (38), while APP is reportedly induced in reactive glial cells (39). Analysis of raw data from SWATH showed that acute kisspeptin stimulation increased GFAP content in the POA, while it decreased MT3 and APP levels. In addition, 2D-DIGE proteomics identified GFAP as one of the differentially expressed proteins related to synaptic plasticity, whose levels were also altered in response to Kp-10. These data collectively point toward an effect of kisspeptin on astroglial cells. This was further documented by the ability of i.c.v. Kp-10 to increase gene/protein expression levels not only of *Gfap*/GFAP, but also vimentin, another astrocyte marker (40), in the POA of mice. To our knowledge, this is the first evidence supporting the capacity of kisspeptins to modulate nonneuronal brain cells like astrocytes. Previous data highlighted the participation of glial cells, and particularly astrocytes, in the control of GnRH neurosecretion, mainly via the release of bioactive molecules such as PGE_2_ and other mechanisms, including juxtacrine interactions with GnRH neurons (13, 41). Yet, no evidence had been presented on a role of astroglial cells as transducers of kisspeptin effects on GnRH neurons or any other brain target/function.

Admittedly, our proteomic data did not conclusively prove direct kisspeptin actions in astrocytes. Compelling evidence for such a putative kisspeptin pathway was provided by a combination of expression and functional studies in control rats and mice. Expression of *Kiss1r*, but not of *Kiss1,* was demonstrated in primary cultures of astrocytes from both rodent species. Notably, similar *KISS1R* expression was also detected in human astrocytes, suggesting the potential conservation of this pathway. Phosphorylation of ERK1/2, a canonical element of the kisspeptin signaling pathway, was consistently induced by kisspeptin in rat and mouse astrocytes in culture; increased phosphorylation of AKT was observed also in rat astrocyte cultures 10 min after kisspeptin stimulation. While it can be argued that astrocyte cultures from neonatal rodents might not fully recapitulate all features of later developmental periods, these have proven valid to evaluate key aspects of astrocyte physiology (42). Additionally, colocalization of *Kiss1r* expression with GFAP and S100-β, as canonical markers for detection of astrocyte processes and cell bodies, was conclusively documented in adult mice in vivo, in key brain areas for reproductive control, such as the OVLT, which holds a large proportion of GnRH neurons, as well as the AVPV and ARC. Enrichment of *Kiss1r*/GFAP/S100-β colocalization was observed in these GnRH-/Kiss1-abundant areas, compared with the cortex, suggesting that kisspeptin signaling in astrocytes might display some degree of region specificity; a contention further supported by the fact that responses to Kp-10 in terms of ERK1/2 phosphorylation were not detectable in cortical astrocyte cultures. Expression of *Kiss1r* gene in astrocytes from adult male and female mouse brains further supported a tenable kisspeptin signaling pathway in these glial cells under physiological conditions. This contention is reinforced by the close appositions between astrocytic (GFAP-positive) projections and Kiss1 neurons found in key hypothalamic areas, as the ARC and AVPV, providing the potential anatomical substrate for the source of kisspeptin input to astrocytes. Further, the dynamic changes in the number of appositions between kisspeptin fibers and GnRH neurons across the ovarian cycle seemingly also engaged changes in the interplay with GFAP-positive processes, suggesting a role of astrocytes in the modulation of such Kiss1-GnRH neuronal interactions. However, our anatomical and FACS analyses also conclusively documented expression of *Kiss1r* in brain areas, such as the cortex, not primarily involved in reproductive control, whose role and putative physiological relevance warrant independent investigation.

The physiological role of direct kisspeptin actions in astrocytes was further addressed by functional genomic analyses, assessing reproductive and metabolic markers in our mouse model of congenital ablation of *Kiss1r* in GFAP-expressing cells. While the use of *Gfap*-driven Cre mouse lines might cause targeting of some neuronal lineages due to potential Cre expression in radial glial cells (43), congenital ablation was posed with obvious advantages in our model, as we intended to explore early maturational events, including puberty, for which inducible models using tamoxifen are not applicable or have important limitations. Further, constitutive Gfap-Cre mouse lines have been recently used for selective astrocyte activation using optogenetics (44) and to successfully target astrocytes in different models, including constitutional ablation of insulin receptors (20), connexin 43 (45), or interleukin-6 (46) in *Gfap*-expressing cells in which specific astrocyte targeting was thoroughly documented using reporter mouse lines. In our G-KiR-KO model, effective recombination was demonstrated in the vast majority (approximately 90%–95%) of astrocytes in relevant hypothalamic areas, including the ARC, AVPV, and OVLT, using a genetic reporter model for astrocyte labelling and a combination of 2 astrocytic markers. In contrast, only 25%–30% of nonastrocytic, NeuN-positive cells in these areas showed GFP-immunoreactivity, supporting a clear astrocyte-preferential recombination in our G-KiR-KO line. In good agreement, primary astrocyte cultures from conditional null mice displayed low to negligible levels of *Kiss1r* mRNA expression. Collectively, these data confirm the validity of our model.

Effective ablation of *Kiss1r* in GFAP-positive cells failed to impact the timing of puberty in both sexes, which was associated with grossly preserved fertility. However, G-KiR-KO mice displayed alterations in the patterns of LH responses to kisspeptin stimulation and LH secretory profiles. These phenotypic features are different from those of global *Kiss1r*-KO mice (27), which suffer from severe central hypogonadism, or mice with conditional inactivation of *Kiss1r* in GnRH neurons, which phenocopy global *Kissr1*-KO mice (4, 5), or in POMC neurons, which are devoid of a detectable phenotype (47) (Supplemental Table 1). Notably, LH responses to an i.c.v. bolus of Kp-10 were not only preserved but were even enhanced in mice with *Kiss1r* ablation in astrocytes, a phenomenon that was not observed in global or GnRH-specific *Kiss1r* KO (5, 27) and was more evident in females than in males, suggesting possible sex difference in the effects of kisspeptin signaling in astrocytes. This finding argues against the possibility of a general recombination affecting neuronal cells driven by our Gfap-Cre model, as this would have resulted in ablation of *Kiss1r* from GnRH neurons and, hence, elimination of LH responses to kisspeptin. Conversely, our data strongly suggest that kisspeptin signaling in astrocytes might play an acute suppressive role in modulating kisspeptin effects on GnRH neurons. Considering the high potency of the direct effects of kisspeptin on GnRH neurons, such a loop would operate as a mechanism for self restraining the magnitude of GnRH pulses after kisspeptin stimulation. In line with this repressive role, congenital elimination of *Kiss1r* from astrocytes resulted in upregulation of the expression of genes encoding inducible factors involved in PGE_2_ synthesis, namely COX-2 and mPGES-1, suggesting that G-KiR-KO mice have increased astrocyte production of this PG, which is a major stimulatory signal for GnRH neurons (17). This could explain the enhanced acute LH responses to Kp-10 in mice with ablation of *Kiss1r* in astrocytes. Furthermore, the reduction of *Cox-1* expression in astrocytes from G-KiR-KO mice could also contribute to enhanced PGE_2_ synthesis, as downregulation of *Cox-1* has been previously shown to facilitate PGE_2_ production in astrocytes (48). These changes did not affect other constitutive elements of the PGE synthesis pathway. Intriguingly, the metabolic hormone ghrelin has been shown to induce opposite (stimulatory) responses in terms of PGE_2_ synthesis in astrocytes as a means to modulate other hypothalamic circuits, such as AgRP neurons (19). Since ghrelin may suppress hypothalamic *Kiss1* expression (49), it is plausible that, at least partially, this stimulatory effect could stem from ghrelin’s capacity to reduce the inhibitory tone of kisspeptin on the PGE_2_ synthetic pathway in astrocytes.

Female G-KiR-KO mice displayed differences in the pattern of LH pulsatility, defined by lower basal LH levels and total LH secretion, despite a trend toward a higher number of LH pulses. These secretory alterations were coupled to changes in the number of appositions between astrocytes and GnRH neurons, which were increased in G-KiR-KO mice. Recent evidence has documented that ARC Kiss1 neurons are a central component of the GnRH pulse generator (7); our present findings suggest that, in addition to direct effects on GnRH dendrons, kisspeptin actions on astroglial cells may contribute to proper shaping of the secretory profiles of GnRH, denoted by changes in LH pulsatility, as surrogate marker of GnRH. While the trend to an increased number of LH pulses in G-KiR-KO mice is compatible with the proposed role of kisspeptin actions in astrocytes, as a putative self-restraint mechanism for kisspeptin-induced GnRH secretion, the suppression of basal LH levels and LH secretory mass likely reflects some partial desensitization due to excessive kisspeptin stimulation. This might also be linked to changes in astrocyte-GnRH neuronal interactions, suggestive of perturbations of normal GnRH neuronal ensheathment after ablation of *Kiss1r* in astrocytes. It must be stressed, though, that these changes did not translate into overt alterations of adult reproductive function in basal conditions*,* except for a modest shortening of the length of the ovarian cycle, suggesting some degree of redundancy of kisspeptin regulatory action on astrocytes regarding preservation of fertility, as was described previously for *Kiss1* expression itself (50). Of note, despite abundant coexpression of *Kiss1r* and GFAP/S100-β in the AVPV, where Kiss1 neurons involved in preovulatory surge are located, the magnitude of estrogen-primed LH surges was preserved in G-KiR-KO female mice. Recent evidence has suggested that positive feedback effects of estradiol also involve stimulation of neuroprogesterone synthesis in hypothalamic astrocytes, which seems to be required for activation of AVPV Kiss1 neuron population to drive the preovulatory LH surge (51). Our findings in G-KiR-KO mice suggest that kisspeptin signaling in astrocytes is dispensable for such positive feedback action of estrogen and point out a role in the modulation of pulse, but not surge mode, of GnRH secretion. In good agreement, no changes in gene expression of key steroidogenic factors were detected in astrocytes from G-KiR-KO mice. Likewise, ablation of *Kiss1r* from astrocytes did not perturb markers of proliferation, differentiation, or cell adhesion, suggesting that these key functions are not physiologically modulated by direct kisspeptin actions.

Compelling evidence has recently documented that astrocytes play a fundamental role in the brain mechanisms governing energy homeostasis (52, 53), and conditions of metabolic stress, such as exposure to a HFD, are known to cause reactive changes in astrocytes, which are putatively involved in mediating at least part of the metabolic deregulations associated with obesity (54). Moreover, key hormones, such as leptin and ghrelin, also endowed with important reproductive roles (55), are known to modulate metabolic homeostasis via direct actions in astrocytes (18, 19, 56). In this context, we considered it relevant to evaluate the reproductive phenotype of G-KiR-KO mice under an obesogenic diet, which revealed a sex-biased effect. While HFD exposure caused an acceleration of puberty onset in control female mice, in line with previous reports in rats (57), conditional ablation of *Kiss1r* in astrocytes largely prevented this effect, suggesting that astrocytic responses to a HFD, putatively involved in advancing pubertal onset in the female, are partially prevented in the absence of kisspeptin signaling in astrocytes. In contrast, no differences in pubertal timing were noted between male control and G-KiR-KO mice fed a HFD. Of note, the effect observed in pubertal G-KiR-KO females was more evident in terms of the VO, which denotes initiation of puberty, than in terms of age of the FE, an index of ovulation and attainment of fertility, suggesting that perturbed astrocyte function caused by *Kiss1r* ablation is possibly more relevant in terms of pubertal activation than completion. Female G-KiR-KO mice also displayed alterations of estrous cyclicity under HFD conditions, with longer cycles and shorter periods at the ovulatory phase, estrus, a phenomenon that was not detected under normal feeding. Similarly, a HFD aggravated the changes in LH pulsatility observed in G-KiR-KO mice. Altogether, these findings strongly suggest that females devoid of kisspeptin signaling in astrocytes are more susceptive to the deleterious effects of a HFD on the adult gonadotropic axis. Considering the suspected dual impact of obesity on Kiss1 neurons (3), with initial over activation followed by long-term suppression, our data suggest that this astrocyte regulatory circuit, parallel to the direct effects of kisspeptins on GnRH neurons, contributes to adaptive responses of the reproductive axis to obesogenic stressors, both during puberty and adulthood, mainly in females.

In terms of metabolic profiles, male and female G-KiR-KO mice failed to show overt differences in adult BW or composition versus control animals fed either a normal diet or a HFD. However, G-KiR-KO mice displayed modestly improved glycemic responses to glucose overload without consistent changes in insulin sensitivity. This observation suggests that kisspeptin signaling in astrocytes may participate in the control of glucose homeostasis, with a predicted function as a factor favoring (modest) glucose intolerance. While the mechanisms for such a phenomenon are yet to be clarified, this putative function appears to be more evident in males than in females, as, in males fed a chow diet the integral GTT responses were significantly diminished, as an index of improved glucose tolerance. Similar trends were detected in G-KiR-KO males fed HFD. Opposite alterations, namely, a worsening of glucose tolerance, have been reported in mice with conditional ablation of insulin receptors in GFAP-expressing cells (58), suggesting an opposite role of insulin versus kisspeptin signaling in astrocytes in the control of peripheral glucose homeostasis.

In the last decades, the pivotal role of glial cells, and particularly of astrocytes, in the central modulation of reproductive function has been defined, with a prominent role in the neurohormonal regulation of GnRH neurosecretion. In addition, insulin has been recently shown to target astrocytes to putatively modulate GnRH neurons, and, thereby puberty onset and gonadal function (20). A recent report has documented, using pharmacogenomics, that global activation of GFAP-positive cells in the vicinity of GnRH neurons stimulated GnRH neuronal firing and LH secretion (59), therefore confirming the prominent functional role of astrocytes in GnRH control. Our present data disclose an additional, previously unnoticed regulatory pathway involving direct kisspeptin actions in astrocytes, which is likely to operate as self-restraint mechanism for the potent releasing effect of kisspeptins on GnRH secretion, relevant for shaping pulsatile GnRH secretion. This nonneuronal signaling pathway of kisspeptins in the brain may also mediate at least part of the adaptive reproductive responses to metabolic stressors, such as an obesogenic diet.

## Methods

Detailed description of Methods can be found at Supplemental Methods.

### Sex as biological variable

Studies were implemented in male and female mice to explore potential sex-related differences. Initial exploratory analyses were done in males, but based on sex differences found, more in-depth characterization of kisspeptin signaling in astrocytes was conducted in females.

### Animals

Mice were housed in the Experimental Animal Service of the University of Córdoba or animal facilities of the Universities of Cambridge or Lille. All animals were maintained at 12-hour light/dark cycle at standard temperature (22 ± 2°C) with ad libitum access to standard laboratory mice chow (A04, Panlab) and water, unless mentioned otherwise. The day the litters were born was considered postnatal day 1 (PND1); animals were weaned at PND23. For the diet-induced obesity studies, mice were fed from weaning onward with high-fat diet (HFD, D12331; Research Diets) with 58%, 17%, and 25% calories from fat, proteins, and carbohydrates, respectively.

### Experimental designs

#### Hypothalamic proteomic profiles after acute central administration of Kp-10 in Kiss1-KO mice.

We conducted proteomic analyses of hypothalamic (POA) tissues of adult Kiss1 KO mice (*n* = 9) after i.c.v. injection of an effective dose of Kp-10 (50 pmol). Adult Kiss1-KO male mice (*n* = 6) i.c.v. injected with vehicle (Veh; 0.9% saline) served as controls. To avoid the potential confounding factor of kisspeptin-induced changes in testosterone levels, mice were orchidectomized 3 weeks before Kp-10 injection. Animals were euthanized 60 minutes after Kp-10 administration, and the POA was excised and processed for proteomic determinations (nano-HPLC associated to triple-TOP equipped with SWATH acquisition or 2-DIGE).

#### Effect of acute central administration of Kp-10 on astrocyte glial markers in Kiss1 KO mice.

RT-qPCR and Western blot analyses were performed to assess the effect of central Kp-10 injection on the astroglial markers GFAP and vimentin. Adult Kiss1 KO male mice were i.c.v. injected with Kp-10 or vehicle (*n* = 3–4 per group). After 60 minutes, animals were euthanized and the POA was excised and processed for analysis.

#### Kiss1r expression in primary astrocyte cultures and functional studies.

To assess whether *Kiss1r* is expressed in astrocytes and, subsequently, whether it is functional, primary astrocyte cultures from the hypothalamus of neonatal rats and mice were generated. *Kiss1r* expression was assessed by RT-qPCR, which was also applied to human astrocyte cultures. Once expression of *Kiss1r* was demonstrated, we conducted functional studies. Astrocyte cultures from neonatal rats were incubated with Kp-10 or vehicle (veh) at different times (1, 10, and 30 minutes). Functional studies were conducted also in astrocyte cultures from neonatal mice, incubated during 10 minutes with Kp-10, based on our results from rat cultures. Cortical astrocyte cultures were also included to compare effects in astrocytes from different brain locations. Mouse astrocyte cultures were also incubated with epidermal grown factor (EGF; 50 ng/mL, Gibco), as positive control (60). Finally, astrocyte cultures from G-KiR-KO mice were incubated with Kp-10 (10^–8^ M), at 1 and 10 minutes. Astrocyte cultures from control mice were used as a positive control.

#### Colocalization of Kiss1r and GFAP/S100-β in mouse brain and Kiss1r expression in isolated astrocytes.

To evaluate the astrocyte expression of *Kiss1r* in vivo, we first used RNAscope to combine RNA ISH with immunofluorescence (the latter for GFAP and S100-β detection, as canonical astrocyte markers). Brain sections from 4 adult female control mice at diestrus were incubated with the probes for *Kiss1r* (revealed in green) and *GnRH* (revealed in far red). Thereafter, IHC detection of GFAP (revealed in cyan) and S100-β (revealed in magenta) was performed. For each animal, 2 slides were taken: 1 covering the OVLT and AVPV, and the second including the ARC and cortex. In parallel, astrocytes were isolated from the POA, MBH, and cortex from adult male (*n* = 6) and female (*n* = 6) mice, using FACS, and *Kiss1r* expression was assessed by qPCR.

#### Assessment of appositions between GFAP-positive astrocytes and Kiss1 and GnRH neurons.

Double IHC analyses were conducted in diestrus female mice (*n* = 2) for assessing whether there are close appositions between Kiss1 neurons (revealed in magenta) and GFAP-expressing astrocytes (revealed in green) in the ARC and AVPV. In addition, triple immunofluorescence detection was conducted in cyclic control female mice to interrogate the intimate appositions between Kiss1 and GnRH neurons with GFAP-expressing astrocytes across the estrous cycle.

#### Phenotypic evaluation of sexual maturation, estrous cyclicity, and fertility in adult G-KiR-KO mice.

3-week-old control male (*n* = 20) and female (*n* = 16) mice, and age-paired G-KiR-KO male (*n* = 12) and female (*n* = 16) mice, were checked for phenotypic markers of puberty (61). In adulthood, control (*n* = 7) and G-KiR-KO (*n* = 11) female mice were monitored daily to assess estrous cyclicity. Virgin control (*n* = 4) and G-KiR-KO (*n* = 5) mice were crossed with control males to assess fertility rates and breeding intervals.

#### Pharmacological studies in adult G-KiR-KO mice.

LH responses to Kp-10 were studied in both G-KiR-KO male (*n* = 8) and female (*n* = 5) mice. Control (males, *n* = 4; females, *n* = 10) and G-KiR-KO mice of both sexes were i.c.v. injected with Kp-10 (50 pmol). Blood samples were collected before (basal) and 15, 30, and 60 minutes after injection.

#### Assessment of pulsatile and surge LH secretion in G-KiR-KO female mice.

Assessment of pulsatile LH secretion was conducted in control (*n* = 10) and G-KiR-KO (*n* = 5) female mice. The LH surge profiles were analyzed also in control (*n* = 5) and G-KiR-KO (*n* = 3) female mice, as described in Supplemental Methods.

#### Activation of GnRH neurons and astrocyte appositions after kisspeptin stimulation in G-KiR-KO mice.

To evaluate whether the lack of kisspeptin signaling alters astrocyte appositions to GnRH neurons and/or their activation following kisspeptin stimulation, adult control (*n* = 5) and G-KiR-KO (*n* = 5) female mice at diestrus were i.p. injected with an effective dose of Kp-54 (1 nmol). Activation of GnRH neurons was assessed by IHC detection of cFos, in line with previous references showing higher efficiency of Kp-54 to induce cFos expression (30). In addition, double IHC was applied to label GFAP and GnRH signals in the brains of these animals.

#### Analysis of metabolic and reproductive phenotypes of G-KiR-KO mice after a HFD.

G-KiR-KO mice and their controls were fed a HFD from weaning onward for generation of diet-induced obesity. Three-week-old male (*n* = 35) and female (*n* = 16) controls, as well as G-KiR-KO male (*n* = 17) and female (*n* = 9) mice fed a HFD were checked for phenotypic markers of puberty on a daily basis, as described for mice fed a chow diet. Adult virgin control (*n* = 7) and G-KiR-KO (*n* = 7) female mice, fed a HFD, were monitored daily for at least 3–4 weeks to characterize estrous cyclicity. In addition, adult male (*n* = 9) and female (*n* = 13) controls and male (*n* = 14) and female (*n* = 10) G-KiR-KO mice on a HFD were i.c.v. injected with Kp-10 (50 pmol) and LH secretory responses were monitored. Additional groups of control (*n* = 6) and G-KiR-KO (*n* = 8) female mice, fed a HFD for 2 months, were subjected to analysis of LH pulsatility. Body composition analyses were also conducted in adult (4-month-old) male (*n* = 20) and female (*n* = 10) controls, and male (*n* = 13) and female (*n* = 6) G-KiR-KO mice fed a HFD. Finally, GTT and ITT were conducted in adult male (*n* = 10) and female (*n* = 10) controls and male (*n* = 10) and female (*n* = 9-10) G-KiR-KO mice fed a HFD.

#### Gene expression analyses in primary astrocyte cultures from G-KiR-KO mice.

To evaluate whether ablation of *Kiss1r* in astrocytes alters the expression of key elements in astrocyte physiology, gene expression analyses were applied to primary cultures of astrocytes from control and G-KiR-KO mice. The genes analyzed and their corresponding functional pathways are as follows: *Sox-2* and *Nanog* (progenitor and differentiation markers); *Ki67* and*Cdk2* (cell proliferation and migration); *Tspo*, *Star*, *P450scc*, *Hsd3b1*, and *P450arom* (steroidogenic pathway factors); *SynCam1*, and *Ncam1* (cell adhesion and glia-to-GnRH neuron interaction factors); and *Cox-1*, *Cox-2*, *mPges*, *Pges2,* and *cPges* (PG synthesis pathway).

### Statistics

Statistical analyses were performed using Prism software (GraphPad Prism). All data are presented as mean ± SEM. Group sizes are denoted in the Methods section and/or figure legends for each experiment. Sample sizes were defined in line with our previous experience in experimental studies using rodent species to evaluate the neuroendocrine regulation of puberty and adult reproductive function (62), assisted by previous a priori power analyses using dedicated software (e.g., GRANMO, https://apisal.es/Investigacion/Recursos/granmo.html) and values of SD that we usually obtain when measuring analogous parameters. These predictions defined that the selected sample sizes would provide at least 80% power to detect effect sizes using the tests indicated above, with a significance level of 0.05. Nonetheless, according to standard procedures, more complex molecular and histological analyses were implemented in representative subsets of randomly assigned samples from each group. Unless otherwise stated, unpaired 2-tailed Student’s *t* tests were applied for assessment of differences between 2 groups, while 1- or 2-way ANOVA (as indicated in figure legends) followed by post hoc Bonferroni tests were applied for comparisons of more than 2 groups. A *P* value less than 0.05 was considered significant, and different letters and/or asterisks have been used to indicate statistical significance. As general principle, the investigators directly performing animal experimentation and analyses were not blinded to the group allocation, but primary data analyses conducted by senior authors were conducted independently to avoid any potential bias.

### Study approval

Unless otherwise stated, the experiments and animal protocols were approved by the Ethical Committee of the University of Córdoba and Junta de Andalusia; animal experiments were conducted in accordance with European Union normative for the use and care of experimental animals (EU Directive 2010/63/UE, September 2010). In addition, for experiments involving Kp-10 i.c.v. injection in Kiss1-KO mice, animals were killed in accordance with the UK Home Office regulations under the Animal (Scientific Procedures) Act of 1986. Establishment, breeding, and care of this mouse line were approved by a Local Ethics Committee at Cambridge University and performed under authority of a Home Office License (UK). Finally, some of the studies — those involving primary cultures of mouse astrocytes and RNAscope analyses in brain tissue sections and FACS isolation of mouse astrocytes — were conducted under approval of the Institutional Ethics Committee for the Care and Use of Experimental Animals of Lille University (APAFIS no. 2617-2015110517317420 v5). The studies on primary cultures of astrocytes from cortical and hypothalamic tissues microdissected from human fetuses were approved by the French Agency for Biomedical Research (France; protocol no. PFS16-002).

### Data availability

The authors declare that the data supporting the findings of this study are included in this article and its supplemental information files, including the Supplemental Data Values file. All relevant source data are provided at the following DOI: https://doi.gin.g-node.org/10.12751/g-node.z9q4zs/ Any additional information will be made available from the corresponding authors, upon request.

## Author contributions

ET was responsible for experimental studies, primary analysis and evaluation of data, draft of figures and manuscript. GP was responsible for RNAscope and immunohistochemical analyses, with the assistance of ACR. MGR was responsible for in vivo experiments, including characterization of G-KiR-KO mice and LH pulsatility, with the assistance of CPL and SL. ACFF was responsible for astrocyte cultures from G-KiR-KO mice and gene expression analyses thereof. IV was responsible for generation of mouse models and expression/functional studies. AL was responsible for optimization of FACS and gene expression analyses thereof. TLH and FN were responsible for conception/interpretation of triple labeling immunocytochemical studies conducted by MS. SHY and SMM was responsible for initial functional studies in Kiss1-KO mice and protein/RNA analyses. MJST, VMN, and RP were responsible for immunohistochemical analyses and protein analyses. JR was responsible for in vivo studies and discussion of data. JA and JAC were responsible for generation/analysis of rat astrocyte cultures and discussion of data. RML was responsible for astrocyte cultures and gene expression thereof. AS and VP were responsible for design/implementation of mouse astrocyte cultures, RNAscope and FACS and discussion of data. WHC was responsible for Kiss1-KO studies and assistance in study design. MTS and ARR designed and cosupervised the whole study and analyzed and discussed the data. ARR had leading roles in proteomic analyses, while MTS was project leader and senior responsible for final preparation of the manuscript. All authors take full responsibility of the work. MTS and ARR are both senior/corresponding authors of this study.

## Supplementary Material

Supplemental data

Unedited blot and gel images

Supporting data values

## Version 1 06/11/2024

In-Press Preview

## Version 2 08/01/2024

Electronic publication
