# Supplementary material for: Kisspeptin signaling in astrocytes modulates the reproductive axis
Source: J Clin Invest. 2024 Jun 11;134(15):e172908. doi: 10.1172/JCI172908 (PMC11291270; doi:10.1172/JCI172908)
Supplement: Supplemental data [file jci-134-172908-s170.pdf]

SUPPLEMENTAL MATERIALS

Supplemental (Extended) Methods

Supplemental Figures and Tables

Kisspeptin signaling in astrocytes modulates the reproductive axis

E. Torres[1,2,3], G. Pellegrino[4,†], M. Granados-Rodríguez[1,2,3,†], A.C. Fuentes-Fayos[1,2,3], I. Velasco[1,2,3], A. Coutteau-Robles[4], A. Legrand[4], M. Shanabrough[5], C. Perdices-Lopez[1,2,3], S. Leon[1,2,3], S.H. Yeo[6], S.M. Manchishi[6], M.J. Sánchez-Tapia[1,2,3], V.M. Navarro[9], R. Pineda[1,2,3], J. Roa[1,2,3], F. Naftolin[7], J. Argente[8,10,11], R.M. Luque[1,2,3,8], J.A. Chowen[8,10], T.L. Horvath[5], V. Prevot[4], A. Sharif[4], W.H. Colledge[6], M. Tena-Sempere[*,1,2,3,8], A. Romero-Ruiz[*,1,2,3]

[†] *Equally contributed and should be considered joint second authors*

* *Equal senior and corresponding authors*

[1]Instituto Maimónides de Investigación Biomédica de Córdoba (IMIBIC), Córdoba, Spain; [2]Department of Cell Biology, Physiology and Immunology, University of Córdoba, Córdoba, Spain; [3]Hospital Universitario Reina Sofía, Córdoba, Spain; [4]University of Lille, Inserm, CHU Lille, Laboratory of Development and Plasticity of the Neuroendocrine Brain, Lille Neurosciences & Cognition, UMR-S1172, Lille, France; [5]Program in Integrative Cell Signaling and Neurobiology of Metabolism, Department of Comparative Medicine, Yale University School of Medicine, New Haven, CT, USA; [6]Reproductive Physiology Group, Physiology, Development and Neuroscience, University of Cambridge, Cambridge, UK; [7]Centro de Fecundacion In Vitro Angela Palumbo (FIVAP), La Laguna, Spain; [8]CIBER Fisiopatología de la Obesidad y Nutrición, Instituto de Salud Carlos III, Madrid, Spain; [9]Division of Endocrinology, Diabetes, and Hypertension, Brigham and Women's Hospital, Harvard Medical School, Boston, USA; [10]Department of Endocrinology, Hospital Infantil Universitario Niño Jesús, Instituto de Investigación La Princesa, and IMDEA Food Institute, CEI UAM + CSIC Madrid, Madrid Spain; [11]Department of Pediatrics, Universidad Autónoma de Madrid, Madrid, Spain.

**** Corresponding authors:*** Manuel Tena-Sempere (fi1tesem@uco.es); and
Antonio Romero-Ruiz (b72rorua@uco.es)
Department of Cell Biology, Physiology & Immunology
Faculty of Medicine, University of Córdoba
Avda. Menéndez Pidal s/n. 14004 Córdoba, SPAIN

Extended Methods

Kiss1 KO mice. Proteomic analyses were conducted in hypothalamic samples encompassing the POA from mice with congenital inactivation of Kiss1, after icv stimulation with a bolus of Kp-10. This model, name Kiss1 KO herein, was generated by replacement of *Kiss1* gene with an internal ribosome entry site (IRES)-LacZ reporter gene, as described in detail elsewhere (1).

Generation of G-KiRKO mouse line. To ablate kisspeptin signaling in astrocytes, conditional ablation of *Kiss1r* from GFAP-positive cells was accomplished using Cre-loxP technology. A well-validated Gfap-Cre mouse line, which express Cre recombinase under the control of the human glial fibrillary acidic protein (hGFAP) promotor (2), generously provided, under appropriate MTA, by A. Pascual and R. Pardal, Univ. of Seville, Seville, Spain, was crossed with Kiss1r^{loxP} mice, obtained from Lexicon Pharmaceuticals (The Woodlands, Texas, USA), in which the *Kiss1r* gene is flanked by *loxP* sites (3). The resulting double transgenic line was named G-KiRKO, for **G**FAP cell-specific **Ki**sspeptin **R**eceptor **KO**).

Generation of GFAP-YFP reporter mouse line. To validate our G-KiRKO model, we generated a reporter mouse line, termed herein GFAP-YFP, by crossing the Gfap-Cre transgenic line, described above, with the YFP^{loxP} mouse line (B6.129X1-Gt(ROSA)26Sor$^{tm1(EYFP)Cos}$/J; JAX) which contains an enhanced yellow fluorescent protein (YFP) gene blocked by a *loxP*-flanked STOP sequence.

Genotyping of the G-KiRKO mouse line. For genotyping of G-KiRKO mice, two PCR amplifications per mouse were set: one to detect the wild-type (WT) and/or *loxP* flanked *Kiss1r* allele and another to detect the *Cre* transgene. The Kiss1r-Flox primers (5'- AGCGCAAGGCTCTGAAGCGGC -3' and 5'- CAATGT CGCCTCGGTGGCCAT -3') were designed to produce a 288-bp band to indicate WT allele and a 326-bp band for the *Kiss1r* allele flanked with *loxP* sites. Gfap-Cre primers (5'- ACGGGCACTGTGTCCAG -3' and 5'- TGTTCAGGGATCGCCAG -3') produced a 788-bp band that denotes the presence of the *Cre* transgene. PCR reactions were carried out in a T100™ thermal Cycler (Bio-Rad, Hercules, CA, USA) using the following cycling conditions: denaturing for 5 min at 95°C followed by 30 cycles of denaturing at 95°C for 30 s, annealing at 60°C for 30 s, and extension at 72°C for 10 s. A final extension step at 72°C for 5 min was performed. Samples were analyzed on 2% agarose 1X TBE (TBE 10X: 10% Tris-HCl, 5% boric acid and 4% EDTA) gels. For visualization of electrophoresed PCR products, gels were stained with RedSafe™ Nucleic Acid Staining Solution (INtRON), and digital images were captured in a Molecular Imager® Gel Doc™ XR System (Bio-Rad).

Genotyping of the GFAP-YFP reporter mouse line. For this genotyping, three PCR amplifications per mouse were required: one to detect the WT allele, another for the mutant allele and the last one for detecting the *Cre* transgene. The EYPF WT primers (5'- AAAGTCGCTCTGAGTTGTTAT -3' and 5'- GGA GCGGGAGAAATGGATATG -3') produce a 600-bp amplicon to indicate the WT allele. The EYFP mutant primers (5'- AAAGTCGCTCTGAGTTGTTAT -3' and 5'- AAGACCGCGAAGAGTTTGTC -3') produce a 320-bp amplicon that indicates EYFP construction allele. As described in the G-KiRKO mice model, Gfap-Cre primers were used to detect the presence of the *Cre* transgene (788-bp band). PCR reactions were carried out as previously described.

Validation of G-KiRKO mouse line. To provide a general validation of our G-KiRKO model, three complementary approaches were carried out. First, effective recombination denoting Cre activity was assessed by PCR in genomic DNA of different tissues from G-KiRKO mice. To this end, the pair of primers 5'- AGCGCAAGGCTCTGAAGCGGC -3' and 5'- AACAACCCGTCGGATTCTCCG - 3' were used to detect the presence of the recombination event (750-bp amplicon). Second, to assess whether Cre recombinase is functional in astrocytes, we used a reporter mouse line generated by crossing Gfap-Cre transgenic mice with YFP^{loxP} reporter mice, in which the expression of YFP, the reporter signal, is blocked by a *loxP*-flanked STOP sequence and hence becomes expressed only after Cre-mediated recombination. Triple immunohistochemistry was used to co-localize GFAP and the cytosolic glial marker, S100β (4), with YFP (denoted by GFP immunoreactivity). Immunohistochemical analyses were conducted in relevant reproductive hypothalamic areas, namely the ARC, AVPV, OVLT, and confocal images from serial coronal sections obtained throughout the rostro-caudal extent of each nucleus, were analyzed GFAP-YFP female mice (n=4). For quantitative analyses, the GFP channel was first made invisible and GFAP/ S100β -positive cells were counted (as the denominator). Then, the GFP channel was made visible, and those cells co-labeled with GFP and GFAP/ S100β-immunostaining were counted (as the numerator). The percentage of recombination was estimated as the number of GFP-positive cells/total number of GFAP/S100β-positive cells x 100. No GFP signal was detected in controls. Similarly, double immune-histochemistry was applied to co-localize YFP and the neuronal marker, NeuN, in the same model; equivalent quantitative analyses were applied to estimate % of colocalization of GFP and NeuN in the above regions. The last validation approach was the generation of primary astrocyte cultures from hypothalamic samples of newborn (PND-1 up to PND-3) control (n=3-4) and G-KiRKO (n=3) mice, for molecular analyses of *Kiss1r* expression by qPCR.

Intracerebral injections. For proteomic analyses, Kiss1 KO mice were stereotaxically injected with Kp-10, following previously published protocols (1). For analyses of LH responses to kisspeptin stimulation in G-KiRKO mice, intracerebroventricular (icv) injections of Kp-10 were conducted, in line with previous references (3, 5). Cannulas (INTRADEMIC polyethylene Tubing, Becton Dickinson, Sparks, MD, USA) were implanted, 2-3 days before experiments, into the mouse brain at 2 mm depth, with coordinates of 1 mm posterior and 1.2 mm lateral to Bregma according to a mouse brain atlas to target the lateral ventricle. For cannula implantation, mice were anesthetized with isoflurane and after cannulation, the animals were individually housed until the end of the experiment.

Phenotypic evaluation of pubertal maturation and reproductive assessment of adult mice. After weaning, 3-week-old mice were checked for phenotypic markers of puberty onset on a daily basis. Somatic and reproductive indices of pubertal development were monitored including body weight (BW), age of vaginal opening (VO) and balano-preputial separation (BPS), the latter two ones being considered external markers of puberty in female and male rodents, respectively. Once the vaginal opening occurred, vaginal cytology was performed daily to identify the age of the first estrus (FE), a marker of first ovulation in female rodents. Adult virgin female mice were monitored daily by vaginal cytology for at least 3-4 weeks to characterize the estrous cyclicity. In addition, fertility rates were calculated, when relevant, as the percentage of three consecutive pairings that resulted in a litter, for each group. The breeding interval was determined also by subtracting gestational length from the number of days between pairing and birth, and the litter sizes were recorded.

Evaluation of metabolic parameters. For analyzing the body composition, including assessment of fat and lean mass, in awake mice, quantitative magnetic resonance (QMR) using the EchoMRI™ 700 analyzer (Houston, TX, software v.2.0) was performed. To provide reliable, repetitive determinations, device calibration was conducted in every session by replicate measurements of commercial canola oil with a known fat content, following the manufacturer instructions. For GTT and ITT, mice were fasted for 4 h and subsequently received an ip bolus of glucose or insulin, as described in detail elsewhere (6). Glucose concentrations from tail-tip bleeding were monitored before (basal) and 20, 60 and 120 minutes after ip injections, using a handheld glucometer (ACCU-CHECK Aviva; Roche Diagnostics). For integral analysis of plasma glucose concentrations after GTT and ITT, the total area under the curve (AUC) were determined by the trapezoidal method during the 120 min following glucose and/or insulin administration.

Sample collection. For studies involving real-time PCR (RT-qPCR) and Western blot, tissues were dissected out, immediately upon the decapitation of the animals, frozen in liquid nitrogen and stored at -80°C until used for molecular analyses. Within the hypothalamus, preoptic area (POA) was dissected

following the optic chiasm and the intersection to the decussation of the optic nerves. Total RNA was extracted using a commercial kit for RNA extraction (Favorgen Tissue Total RNA Extraction Mini Kit, FATRK001) according to the manufacturer's recommendations. RNA concentration of each sample was determined using Nanodrop ND-1000 v3.5.2 spectrophotometer (Nanodrop Technology®, Cambridge, UK). For the protein extracts, the flow-through obtained from the RNA-binding column of *"Favorgen Tissue Total RNA Extraction Mini Kit"* was collected from each sample and precipitated by adding four volumes of acetone. The protein pellet was resuspended in lysis buffer, containing 7 M urea (Sigma-Aldrich); 2 M thiourea (Sigma-Aldrich); 1 M Tris (Sigma-Aldrich) and CHAPs (PanReac), until used for molecular analyses.

LH Assays - Assessment of acute responses to LH and pulsatile and surge LH secretion

For hormone LH assays, blood samples were obtained from the mouse-tail at 0, 15, 30 and 60 minutes after icv injection of Kp-10. In detail, in each sample, 4 μL of whole blood were diluted in 46 μl of 0.1 M PBS (phosphate buffered saline) with 0.05 % Tween 20, snap-frozen on dry ice and stored at -80°C until assay using a super-sensitive LH ELISA, whose details are described in detail elsewhere (7, 8). Single replicates were assessed due to limited sample volume. The intra- and inter-assay coefficients of variation were 3.6% and 4.4%, respectively. Intra- and inter-assay variability was assessed by determination of reference LH concentrations (0.25 ng/ml) in replicates, repeated within each assay and across separate assays (n=7), respectively.

For LH pulsatile secretion assay, mice were handled daily (5-10 min per animal) and habituated to being restrained in a cardboard tube every day for 3 weeks before blood sampling, as a way to habituate them used to the protocol and prevent the suppressive effect that the stress might have on LH levels during the tail-tip bleeding (9). During blood sample collection, mice were restrained in the same cardboard tube every 5-min interval during the 3 hours of tail-tip bleeding, in line with previous references (7, 10). The number of pulses (peaks) and total mass of LH secreted in peaks, as well as basal LH levels and total mass of secreted LH were measured. LH pulses were defined as those displaying an increase of 125% over basal values, in line with our previous reference (11). Basal LH levels were determined as the average of the 10 lowest LH measurements from each mouse, as set in previously validated protocols (12), whereas the total mass of LH secreted was estimated as the net increment of integral (area under the curve; AUC) LH secretion determined by the trapezoidal method over the 3-hour study period.

For the LH surge assay, mice were anesthetized with isofluorane, subjected to bilateral ovariectomy and supplemented with subcutaneous (sc) capsules (SILASTIC brand silicon tubing) filled with estradiol (5 μg/ml). Nine days later, diestrus mice were given a sc injection of estradiol benzoate (1 μg/ 20 g BW in

100 µl) at 10.00 am to produce elevated proestrus-like E2 levels on the following day. On that day, tail-tip blood samples were taken in the morning (10.00 am), evening (8.00 pm-light on) and evening after lights off (8.20 pm, 8.35 pm and 8.50 pm), as adapted from previously published protocols (13).

Reverse transcription and real-time PCR (RT-qPCR).

Analyses in rodent samples: For removing potential genomic DNA contamination, 0.1-1 µg RNA samples were DNase-treated following manufacturer's instruction (RQ1 RNase-Free DNase. Promega, WI, USA). For intron-containing genes, specific qPCR primers, spanning over intron regions, were designed to avoid genomic DNA contamination. The reverse transcription reaction was prepared using the iScript™ cDNA Synthesis Kit (1708891; Bio-Rad Laboratories Inc., USA). RNA reversed transcribed into cDNA was analyzed by quantitative RT-PCR using specific primers pairs listed in **Suppl. Table S2**. Go Taq qPCR Master mix (A6102; Promega Corporation, USA) was used for RT-PCR, conducted in a CFX96 Touch Real-Time PCR Detection System (Bio-Rad Laboratories Inc., USA). PCR reactions were performed in duplicates for each experimental sample and consisted of 5 µl cDNA, 0.5 µl specific primer forward (Fw), 0.5 µl specific primer reverse (Rv), 6.25 µl Go Taq qPCR Master mix and 2.75 µl of nuclease free water in a final volume of 15 µl. Negative controls for genomic DNA, RT and RT-qPCR were included. The mRNA input values of each target gene were normalized to ribosomal protein *S11* expression, and the resulting values were expressed as fold change above control levels. Calculation of the expression levels of each target was conducted based on the cycle threshold (CT) method. The CT for each sample was calculated using the iCycler iQ™ Real-time PCR detection system software with an automatic fluorescence threshold setting.

Analyses in human astrocyte culture samples: RNA was extracted as follows: 1 ml of Trizol (Life Scientific, Carlsbad CA, USA) was used to lyse cells before adding 100 µl of chloroform (Merck, Darmstadt, Germany) and centrifuging at 12,000 g for 15 minutes at 4°C. The aqueous phase was carefully collected and added to isopropanol at 1:1 before centrifuging at 12,000 g for 10 minutes at 4°C. The aqueous phase was then discarded and the pellet washed in 70% ethanol. After centrifugation at 12,000 g for 5 minutes at 4°C, the pellet was left to air-dry and then diluted in 10 µl DEPC water. The purity and quantity of RNA was determined by UV spectroscopy (Nanodrop 1000, Thermo Scientific, Waltham MA, USA). Messenger RNAs were reverse transcribed using SuperScript® III Reverse Transcriptase (Life Technologies). Real-time PCR was carried out on Applied Biosystems 7900HT Fast Real-Time PCR System using exon-boundary-specific TaqMan® Gene Expression Assays (Applied Biosystems): *Kiss1r* (mouse: Mm00475046_m1 or human: Hs00261399_m1); Control housekeeping genes: *Actb* (mouse: Mm00607939_s1) or *Gapdh* (human: HS02758991_g1). Gene expression data were analyzed using SDS 2.4.1 and Data Assist 3.0.1 software (Applied Biosystems).

Sample collection for immunofluorescence. Mice were anesthetized by injection of ketamine/xylazine and perfused intracardially with 0.9% saline followed by 4% paraformaldehyde (PFA) in PBS pH 7.4. Brains were collected upon the intracardiac fixation of the animals and post-fixed in 4% PFA for 24 hours at 4ºC. Then, the brains were washed in PBS for 24 hours for removing PFA remnants. Thereafter, the brains were dehydrated by immersion in 30% sucrose in PBS for 48 hours at 4ºC and subsequently frozen at - 80°C until further use for immunohistochemistry.

Immunofluorescence. Coronal sections throughout the hypothalamus were cut at 30 µm thicknesses in a 1:3 series using a cryotome (Leica CM1850 UV). The sections were collected in cryoprotectant. For immunohistochemistry, one set of free-floating sections was washed three times (10 min per wash) in Tris-buffered saline (TBS) pH 7.6 at room temperature with gentle agitation. This was followed by a blocking step for 45 min at room temperature with blocking buffer [2% goat or donkey serum (Sigma-Aldrich), TBS, 0.3% Triton-X (PanReac), 0.25% bovine serum albumin (BSA; Sigma-Aldrich) and 0.01% sodium azide (NaN_3; Scharlau)] or after blocking endogenous peroxidases (TBS 1X, methanol and H_2O_2) for 10 min, specifically for detecting kisspeptin protein. After blocking, sections were incubated with the corresponding first antibodies listed in **Suppl. Table S3**, diluted in blocking buffer, for 1h at room temperature and 72 hours at 4°C with gentle agitation. On the next day, sections were washed three times (10 min per wash) in TBS, and incubated with the corresponding secondary antibody, listed in **Suppl. Table S3,** diluted in blocking buffer, for 90 min at room temperature with gentle agitation. After three more washes in TBS (10 min per wash), the sections were mounted on microscope slides, air-dried and cover-slipped with Fluoroshield™ Mounting Medium with DAPI (Sigma-Aldrich, #F6057). For double- and triple-labelling immunohistochemistry, a similar protocol was run. Primary antibodies were raised in different species and incubated at the same time. Secondary antibodies were used in sequential way. For increasing the sensitivity of the kisspeptin antibody detection, an additional incubation step was required by using avidin-biotin complex for 90 min. Finally, it was revealed with Tyramide SuperBoost reagents, after 10 min incubation.

Assessment of appositions between astrocytes and Kiss1 neurons. To interrogate whether Kiss1 neurons and astrocytes maintain an intimate contact in hypothalamic areas key for reproductive control, brains from wild-type diestrus female mice (n=2) were collected, an immunofluorescence was performed, as described above, for detecting kisspeptin (Kp)- and GFAP- immunoreactivity in the AVPV and ARC nuclei. Confocal images from serial coronal sections throughout the rostro-caudal extent of each nuclei were obtained and processed. In addition, 3-D reconstructions were generated using Imaris cell imaging software (Bitplane).

Immunofluorescence image processing. Images were captured with a Zeiss LSM 710 confocal microscope in JPEG format and processed using the open-source image processing software, ImageJ (National Institutes of Health, Bethesda, Maryland). For image quantification, representative images of the areas of interest were delimited using as reference the mouse section from Allen Brain Atlas (14). Both sides of bilateral structures (e.g., ARC) were counted on a similar number of sections per analyzed nucleus and animal, and replicate values were averaged for further determination of group means.

Triple immunofluorescence for kisspeptin, GnRH and GFAP labelling. Adult cyclic, female mice were euthanized for brain collection, at 10:00 am of each day of the estrous cycle, as assessed by vaginal smears: metestrus, diestrus, proestrus and estrus. An additional set of mice were euthanized at the afternoon (between 14:00 and 16:00) of proestrus, to capture the initial phase of the pre-ovulatory surge. Mice were transcardially perfused with fixative containing 4% paraformaldehyde, 15% picric acid (by volume) and 0.08% glutaraldehyde in 0.1M phosphate buffer (PB). They were post-fixed overnight in glutaraldehyde-free fixative, then washed several times in PB. Fifty-micron brain coronal sections containing the medial septal nucleus and nucleus of the vertical limb of the diagonal band were cut on a vibrating blade microtome (Leica Biosystems, Danvers, MA). After several washes in PB, free-floating sections were incubated for 30 minutes at RT in 5% normal donkey serum, 0.2% triton X-100 in PB. After a brief wash, sections were incubated overnight at RT in an antibody mixture of mouse anti-GnRH (Millipore-Sigma, Burlington, MA), rabbit anti-Kisspeptin (Millipore-Sigma) plus chicken anti-GFAP (Millipore-Sigma); see **Suppl. Table S3**. Sections were then washed several times in PB followed by incubation (1.5 hours at RT) in a mixture of secondary antibodies containing Alexa 488 donkey anti-mouse IgG + Alexa 647 donkey anti-rabbit IgG + Alexa 594 goat anti-chicken IgY (Thermo Fisher, Waltham, MA); see **Suppl. Table S3**. Sections were then rinsed several times in PB and mounted on slides using Vectashield (Vector Laboratories, Burlingame, CA). Images were acquired using a Leica Stellaris 5 WLL (Leica Microsystems, Buffalo Grove, IL), and colors of different immunolabels were digitally assigned.

Assessment of appositions between astrocytes and GnRH neurons as well as GnRH neuronal activation in G-KiRKO mice in response to Kp-54. To explore whether the lack of kisspeptin signaling in astrocytes modifies the activation of GnRH neurons in response to exogenous peripheral administration of kisspeptin, as well as the potential interactions between astrocytes and GnRH neurons at the level of the POA, diestrus control (n=5) and G-KiRKO (n=5) female mice were ip injected with Kp-54 at the dose of 1nmol in 100 µl saline. Immunohistochemical analyses to detect the early neuronal activation marker, cFos, and GnRH were performed. A second round of immunohistochemical analyses were performed to detect GFAP- and GnRH- positive cells in the same groups of female mice. Images

were acquired using a Leica DM 2500 and colors of different immunolabels were digitally assigned. For image quantification, representative images (n=5-9 sections/mouse) from POA were delimited using as reference the mouse section from Allen Brain Atlas. The percentage of GnRH neurons co-expressing cFos was calculated as the number of co-location between cFos and GnRH neurons divided by the total number of GnRH neurons along the representative images taken (n=5-9/per mouse). The percentage of close interactions was calculated as the number of close proximity between GFAP-positive cells and GnRH neurons either in the soma or GnRH fibers into the POA divided by the total number of GnRH neurons detected in the POA.

Western Blot. The protein concentration of each sample was determined using RC DC™ Protein Assay (Bio-Rad). The absorbance was read at a wavelength of 750 nm in a standard spectrophotometer (Beckman DU530). Briefly, total protein lysates (10 and/or 20 µg) were subjected to SDS-PAGE on 9% polyacrylamide gels, electro-transferred on polyvinylidene difluoride membranes (Millipore) and probed overnight at 4°C in the presence of the corresponding primary antibody **Suppl. Table S4**. For protein detection, horseradish peroxidase-conjugated secondary antibodies, as listed also in **Suppl. Table S4**, were used and chemiluminescence ECL Western Blotting Substrate (Thermo Scientific) was applied. Protein levels were normalized to actin and or β-catenin. Densitometric analysis of protein bands was conducted using ImageJ.

Two-Dimensional Difference Gel Electrophoresis (2D-DIGE) labeling & electrophoresis. The protein extracts obtained from the hypothalamus of Kiss1 KO mice (50 µg) were labelled with 400 pmol of either Cy3 or Cy5 dyes (GE Healthcare) (pH 8.0). A 50 µg protein mix containing an equal amount of each protein extract used in the experiment was labelled with 400 pmol of Cy2 dye (GE Healthcare), to define the internal standard sample. Labelled samples were immediately subjected to isoelectric focusing (IEF). A total 150 µg of labelled protein containing three equal amount extracts (Cy3-labeled sample, Cy5-labeled sample and the internal standard) was complemented with 0.5% IPG buffer (pH 3-11), 0.3% DTT and traces of bromophenol blue and applied on Immobiline™ DryStrip (pH 3-11), 18 cm strips (rehydrated in DeStreak™ Rehydration Solution, GE Healthcare) using Ettan™ IPGphor™ Cup Loading Manifold (GE Healthcare). Focusing was performed in the Ettan™ IPGphr™ IEF System following four steps: 3 hours at 300 V (step and hold), 6 hours at 3000 V (gradient); 3 hours at 8000 V (gradient) and 8000 V until steady state was reached. After isoelectric focusing, the IPG strips were incubated in equilibration solution-1 [50 mM Tris-HCl (Sigma-Aldrich), 6 M urea (Sigma-Aldrich), 30% glycerol (Sigma-Aldrich), 2% SDS (Sigma-Aldrich) and 10 mg/mL DTT (Sigma-Aldrich) (pH 8.8)] and equilibration solution-2 [50 mM Tris-HCl, 6 M urea, 30% glycerol, 2% SDS and 25 mg/mL iodoacetamide (pH 8.8)] for 15 min, respectively, and then applied to 5-20% gradient SDS polyacrylamide gel electrophoresis bands (SDS-

PAGE) using SE-600 electrophoresis system (GE Healthcare). Strips were cut by 1 cm at each end to adjust to SE-600 width. For DIGE analysis, fluorescent gel images were obtained using a Typhoon 9400 Scanner and analyzed with the DeCyder Differential analysis software (GE Healthcare) by applying default parameters. For preparative gel, Immobiline™ DryStrips were rehydrated in the presence of 0.5-1 mg of protein and 0.5% IPG buffer, pH 3-11, 0.3% DTT, and DeStreak reagent (GE Healthcare). Both focusing and second dimension were performed as described above for the analytical gel. The proteins selected were analyzed by MALDI-MS(/MS) compatible with silver (GE Healthcare) or SYPRO (Bio-Rad) staining to allow subsequent protein identification in the MS Unit of the Central Service for Research Support (SCAI) of the University of Córdoba.

SWATH-MS protein quantification. Protein extracts obtained from the hypothalamus of Kiss1 KO mice were precipitated with trichloroacetic acid (TCA)/acetone and solubilized in 0.2% RapiGest SF (Waters, Milford, MA, USA) in 50 mM ammonium bicarbonate. Total protein was measured using the Qubit™ Protein Assay Kit (Thermo Fisher Scientific); 50 µg of protein extract was subjected to trypsin digestion following the protocol described by Ortea and co-workers (15). Samples were analyzed using a SWATH data-independent acquisition (DIA) approach for massive protein quantitation. Equal amount of all samples from each group were pooled and subjected to shotgun analysis to build a peptide spectral library. Each pooled sample was analyzed twice by data-dependent acquisition (DDA) nanoscale liquid chromatography, followed by tandem mass spectrometry (nano LC-MS/MS) runs, using a LC system Ekspert™ nanoLC400 (Eksigent, Dublin, CA, USA) coupled to a Triple TOF® 5600+ (Sciex, Redwood City, CA, USA) mass spectrometer system. A top 65 method (250 ms MS survey scan in the range 350-1250 m/z, followed of 65 MS/MS with 60 ms acquisition time in the range 230-1700 m/z) was applied. This method was selected since, during technical installation by Sciex's specialists, optimization of different parameters allowed maximizing the number of proteins and peptides identified in complex samples. On each run, 1 µg of protein was analyzed using a 120 min gradient from 5 to 30% buffer B (buffer A: 0.1% FA in water; buffer B: 0.1% FA in ACN) at a flow rate of 300 nL/min, with a 25 cm x 75 µm Acclaim PepMap100 column (Thermo Fisher Scientific). Peptide and protein identifications from all the DDA runs together were performed using Protein Pilot software v5.0.1 (Sciex) with a mouse SwissProt concatenated target-reverse decoy database, specifying iodoacetamide as cysteine alkylation. The false discovery rate (FDR) was set to 0.01 for both peptides and proteins, thereby minimizing chances of false positives. Normalization was conducted considering all the samples analyzed and based on Total Area Sums.

The MS/MS spectra of the identified peptides were used to generate a spectral library for SWATH peak extraction using the add-in for PeakView Software v2.1 (Sciex) MS/MSALL with SWATH Acquisition

MicroApp v2.0 (Sciex). Peptides with a confidence score above 99% as reported from Protein Pilot database search were included in the spectral library. Depleted serum samples (1 μg per sample) were individually analyzed by a SWATH method using the same LC-MS system and LC gradient, described above for the generation of the spectral library. A variable SWATH method, consisting of on cycles of a TOF survey MS scan (range: 350-1200 m/z, 50 ms acquisition time) followed by 60 MS/MS scans (range: 230-1500 m/z, 90 ms acquisition time) of variable width windows, covering the 350 to 1200 mass range, was optimized according to the ion density found on the previous DDA runs. To set these variable windows, the SWATH Variable Window Calculator_V1.0.xlsx tool, from Sciex, was used. The cycle time was 5.5 seconds, and the range of gradient and liquid chromatography time used for the construction of the spectral library was maintained.

The targeted data extraction of the SWATH raw runs was performed by PeakView, using the MS/MSALL with SWATH Acquisition MicroApp v2.0 and the spectral library created from the shotgun data, with the following parameters; up to seven peptides per protein and ten fragment ions per peptide; shared and modified peptides excluded; six-minute XIC windows; 20 ppm XIC width; 1% FDR threshold. Quality control procedures were applied between samples, involving running a beta-galactosidase digest, as standard, to permit mass calibration of the equipment, cleaning the column to avoid any carry-over effect, and checking of sensitivity and chromatography retention time. All the SWATH runs were retention time-realigned using peptides from the RePliCal iRT peptides (PolyQuant GmbH, Bad Abbach, Germany), that had been previously spiked on each vial according to the manufacturer's instructions. In the resulting library, there were 2728 proteins plus eleven RepliCal_iRT peptides, used to adjust the retention times. The total number of proteins identified and quantified (including both the not significant and the differentially expressed ones) was 2510, out of the 2728 proteins present in the spectral library. Protein quantitation was calculated by adding the peak areas of the ion fragments of each of the corresponding peptides. The mass spectrometry proteomics data have been deposited to the MassIVE data repository, with the dataset identifier MSV000090770.

Prediction of protein interactions and pathway enrichment analysis. The differentially expressed proteins listed from the SWATH quantitative analysis were selected as the target gene list. Then, the target gene list was imported into STRING for network reconstruction and visualization (16). As the interactions between proteins are a required analysis for proteomics studies, the STRING open database has been used to assemble, evaluate and disseminate protein-protein association information. For this analysis, medium confidence, as the minimum required interaction scored, was selected (0.400). In addition, ClueGO from the most updated version of Cytoscape (17) and STRING database portal were used for the enrichment pathways analyses, where the biological processes and cellular components

working in the target gene list described above were noted. Outputs from the enrichment analysis based on Cellular component GO terms were plotted used *ggplot2* R package.

Sample collection for RNAscope *in situ* hybridization. Brains were collected and fixed in 4% PFA in PBS (pH 7.4) for 48 hours. Tissues were then transferred to PBS containing 20% sucrose until they sunk. Subsequently, tissues were embedded in Tissue Tek® (Sakura Finetek) and frozen in methylbutane chilled with liquid nitrogen. The samples were kept at -80° until processing for *in situ* hybridization.

RNAscope *in situ* hybridization combined with immunohistochemistry. Single-molecule fluorescent *in situ* hybridization was performed on 14-μm brain sections of four adult female mice (C57BL/6) in diestrus, using the RNAscope® Multiplex Fluorescent V2 Assay according to the manufacturer's protocols (Advanced Cell Diagnostics, Inc., Newark, CA, USA), with the following modifications on the pretreatments: 1 hour of post-fixation and 8 min of protease IV. Specific probes were used to detect *GnRH*-I (Probe-Mm-Gnrh1-O1-C3, Cat# 476281-C3) and *Kiss1r* (Probe-Mm-Kiss1r, Cat# 408001) mouse mRNAs. Sections were then processed for immunohistochemistry, as follows: after washing in PBS, sections were incubated for 1 hour at room temperature in blocking solution containing PBS 1X, 0.4% normal donkey serum and 0.3% Triton X-100. Afterwards, sections were incubated with rabbit polyclonal anti-GFAP (Z0334, DakoCytomation, Glostrup, Denmark, 1:100) and anti-S100β (RTU, IR504, DAKO), overnight at 4°C in a humid chamber. After rinsing in PBS, Alexa Fluor 647-conjugated anti-IgG (Molecular Probes, 1:500) for GFAP detection and Alexa Fluor 488-conjugated anti-IgG (Molecular Probes, 1:500) for detecting S100β, diluted in blocking solution, were deposited on sections and incubated at room temperature for 2 hours. After washing in PBS, cell nuclei were stained with DAPI according to the RNAscope assay. Note that for detection of GFAP and S100β in positive control samples, including *GnRH* mRNA detection, both astrocyte markers were revealed with the same secondary antibody, Alexa Fluor 488-conjugated anti-IgG (Molecular Probes, 1:500).

Images acquisition and qualitative analysis of RNAscope samples. Mouse brain tissue was analyzed in coronal sections and mapped using as reference the Allen Brain Atlas. Fluorescent signals of *Kiss1r* and *GnRH* transcripts (*in situ* hybridization) and GFAP and S100β immunofluorescence signals were imaged with 20X objective lens on a Zeiss LSM 710 confocal system. All settings were identical for all images. The color label far red was assigned for *GnRH* mRNA and the color label green was assigned for *Kiss1r* mRNA hybridization signal. The color label magenta was assigned for S100β or combined GFAP/S100β signals, while cyan was assigned for GFAP. The numbers of GFAP/S100β- positive cells co-expressing *Kiss1r* mRNA were quantified in two microscopic fields per brain section using ImageJ.

Astrocyte primary culture. Pooled male and female newborn (PND1-3) G-KiRKO and wild-type mice as well as newborn rats were used to generate astrocyte primary cell cultures. Animals were euthanized and the hypothalamus, limited by the rostral border of the mammillary bodies and the intersection to the decussation of the optic nerves, and cortex tissue were micro-dissected and collected in cold DMEM/F12 medium with L-glutamine (Lonza, #BE12-719F). Mechanical dissociation of hypothalamic tissue was done by gentle scraping using a cell scraper (Sarstedt®) over a 20 or 40 µm mesh. The cell suspension was centrifuged (5 min at 3200 rpm, 4°C) and the resulting cell pellet resuspended with 4 ml in growth medium (DMEM/F12 with L-glutamine; 1% Gentamicin/ Amphotericin (GIBCO, #R01510) and 10% Fetal bovine serum (Sigma-Aldrich). Cells were plated on uncoated T25 flasks and left growing for the first 5 days without changing the medium. After that period, fresh growth medium was added and after 48 hours, the flasks were placed at 300 rpm in a shaker incubator at room temperature for 24 hours (done to remove oligodendrocytes and microglial cells from culture), in keeping with previously validated protocols (18, 19), having a period of incubation at 37 ºC for 1 hour to adjust the temperature and gas content, after 12 hours of shaking. After shaking, the medium was replaced with fresh medium and cells were again incubated in growth medium and changed every 3 days until they reached 75-85% confluence. Once confluence was optimal, astrocytes were washed with PBS, trypsinized, centrifuged (5 min at 1000 rpm) and placed in 6-well cell culture plates, for assessing the astrocytic response to Kp-10, when relevant.

For studies on primary cultures of astrocytes from cortical and hypothalamic, non-pathological human fetuses were obtained at GW9-12 from voluntarily terminated pregnancies after written informed consent from the donors (Gynecology Department, Jeanne de Flandre Hospital, Lille, France). Primary cultures of human astrocytes were prepared as previously described (20, 21).

Isolation of astrocytes using fluorescence-activated cell sorting (FACS). A FACS protocol for isolation of adult astrocytes, based on the use of anti-astrocyte cell surface antigen-2 (Anti-ACSA-2) monoclonal antibody, was applied, following previously published protocols (22). The preoptic region, mediobasal hypothalamus and cerebral cortex of male and female mice in diestrus were micro-dissected and enzymatically dissociated using Papain Dissociation System (Worthington, Lakewood, NJ) to obtain single-cell suspensions. Cells were incubated with the anti-ACSA-2-PE antibody (130-116-244; Miltenyi Biotec) or control isotype (130-113-450; Miltenyi Biotec) at a concentration of 15.10^{-2}g/L. FACS was performed using a SH800S Cell Sorter Cytometer (Sony), and provided proprietary software Cell Sorter Software 2.1.0 was used for data acquisition. The sorting decision was based on measurements of PE fluorescence (excitation: 488 or 561 nm; detection: Filter Set: FL2 585/30nm). A total of 4000 PE-positive cells were sorted per animal and directly lysed into 10 µl of extraction buffer containing 0.1% Triton® X-100 and 0.4 unit/µl RNaseOUT™ (Life Technologies). Kaluza Software 2.1 was used for data analysis.

Quantitative RT-PCR analyses of FACS samples. Messenger RNAs obtained from FACS-sorted cells were reverse transcribed using SuperScript® III Reverse Transcriptase (Life Technologies). A linear preamplification step was performed using the TaqMan®PreAmp Master Mix Kit protocol (Applied Biosystems). Real-time PCR was carried out on Applied Biosystems Step One Plus Real-Time PCR System using exon-boundary-specific TaqMan® Gene Expression Assays (Applied Biosystems): *Kiss1r* (Kisspeptin 1 receptor, Mm00475046_m1); *Gfap* (Mm01253033_m1); *Glast* (*Slc1a3*, Mm00600697_m1); *Connexin-43* (*Gja1*, Mm01179639_s1); *Elavl3* (Mm01151962_m1); *Rbfox3* (Mm01248771_m1); *Aif1* (Mm00479862_g1); *Cd31* (*Pecam*, Mm01242576_m1). The housekeeping genes used were: *Actb* (Mm00607939_s1) and *Gapdh* (Mm99999915_g1). Gene expression data were analyzed following a standardized procedure (23).

References (Extended Methods)

1. d'Anglemont de Tassigny X, Fagg LA, Dixon JP, Day K, Leitch HG, Hendrick AG, et al. Hypogonadotropic hypogonadism in mice lacking a functional Kiss1 gene. *Proc Natl Acad Sci U S A.* 2007;104(25):10714-9.
2. Malatesta P, Hack MA, Hartfuss E, Kettenmann H, Klinkert W, Kirchhoff F, et al. Neuronal or glial progeny: regional differences in radial glia fate. *Neuron.* 2003;37(5):751-64.
3. Garcia-Galiano D, van Ingen Schenau D, Leon S, Krajnc-Franken MA, Manfredi-Lozano M, Romero-Ruiz A, et al. Kisspeptin signaling is indispensable for neurokinin B, but not glutamate, stimulation of gonadotropin secretion in mice. *Endocrinology.* 2012;153(1):316-28.
4. Zhang Z, Ma Z, Zou W, Guo H, Liu M, Ma Y, et al. The Appropriate Marker for Astrocytes: Comparing the Distribution and Expression of Three Astrocytic Markers in Different Mouse Cerebral Regions. *Biomed Res Int.* 2019;2019:9605265.
5. Leon S, Barroso A, Vazquez MJ, Garcia-Galiano D, Manfredi-Lozano M, Ruiz-Pino F, et al. Direct Actions of Kisspeptins on GnRH Neurons Permit Attainment of Fertility but are Insufficient to Fully Preserve Gonadotropic Axis Activity. *Sci Rep.* 2016;6:19206.
6. Velasco I, Leon S, Barroso A, Ruiz-Pino F, Heras V, Torres E, et al. Gonadal hormone-dependent vs. -independent effects of kisspeptin signaling in the control of body weight and metabolic homeostasis. *Metabolism.* 2019;98:84-94.
7. Torres E, Velasco I, Franssen D, Heras V, Gaytan F, Leon S, et al. Congenital ablation of Tacr2 reveals overlapping and redundant roles of NK2R signaling in the control of reproductive axis. *Am J Physiol Endocrinol Metab.* 2021;320(3):E496-E511.
8. Steyn FJ, Wan Y, Clarkson J, Veldhuis JD, Herbison AE, and Chen C. Development of a methodology for and assessment of pulsatile luteinizing hormone secretion in juvenile and adult male mice. *Endocrinology.* 2013;154(12):4939-45.
9. Yang JA, Hughes JK, Parra RA, Volk KM, and Kauffman AS. Stress rapidly suppresses in vivo LH pulses and increases activation of RFRP-3 neurons in male mice. *J Endocrinol.* 2018;239(3):339-50.
10. Czieselsky K, Prescott M, Porteous R, Campos P, Clarkson J, Steyn FJ, et al. Pulse and Surge Profiles of Luteinizing Hormone Secretion in the Mouse. *Endocrinology.* 2016;157(12):4794-802.
11. Franssen D, Barroso A, Ruiz-Pino F, Vazquez MJ, Garcia-Galiano D, Castellano JM, et al. AMP-activated protein kinase (AMPK) signaling in GnRH neurons links energy status and reproduction. *Metabolism.* 2021;115:154460.
12. McQuillan HJ, Han SY, Cheong I, and Herbison AE. GnRH Pulse Generator Activity Across the Estrous Cycle of Female Mice. *Endocrinology.* 2019;160(6):1480-91.
13. Leon S, Fergani C, Talbi R, Maguire CA, Gerutshang A, Seminara SB, et al. Tachykinin Signaling Is Required for Induction of the Preovulatory Luteinizing Hormone Surge and Normal Luteinizing Hormone Pulses. *Neuroendocrinology.* 2021;111(6):542-54.
14. Wang Q, Ding SL, Li Y, Royall J, Feng D, Lesnar P, et al. The Allen Mouse Brain Common Coordinate Framework: A 3D Reference Atlas. *Cell.* 2020;181(4):936-53 e20.
15. Ortea I, Ruiz-Sanchez I, Canete R, Caballero-Villarraso J, and Canete MD. Identification of candidate serum biomarkers of childhood-onset growth hormone deficiency using SWATH-MS and feature selection. *J Proteomics.* 2018;175:105-13.
16. Szklarczyk D, Gable AL, Lyon D, Junge A, Wyder S, Huerta-Cepas J, et al. STRING v11: protein-protein association networks with increased coverage, supporting functional discovery in genome-wide experimental datasets. *Nucleic Acids Res.* 2019;47(D1):D607-D13.
17. Doncheva NT, Morris JH, Gorodkin J, and Jensen LJ. Cytoscape StringApp: Network Analysis and Visualization of Proteomics Data. *J Proteome Res.* 2019;18(2):623-32.
18. Sharif A, Legendre P, Prevot V, Allet C, Romao L, Studler JM, et al. Transforming growth factor alpha promotes sequential conversion of mature astrocytes into neural progenitors and stem cells. *Oncogene.* 2007;26(19):2695-706.
19. Prevot V, Rio C, Cho GJ, Lomniczi A, Heger S, Neville CM, et al. Normal female sexual development requires neuregulin-erbB receptor signaling in hypothalamic astrocytes. *J Neurosci.* 2003;23(1):230-9.
20. Sharif A, Duhem-Tonnelle V, Allet C, Baroncini M, Loyens A, Kerr-Conte J, et al. Differential erbB signaling in astrocytes from the cerebral cortex and the hypothalamus of the human brain. *Glia.* 2009;57(4):362-79.
21. Sharif A, and Prevot V. Isolation and culture of human astrocytes. *Methods Mol Biol.* 2012;814:137-51.

22. Kantzer CG, Boutin C, Herzig ID, Wittwer C, Reiss S, Tiveron MC, et al. Anti-ACSA-2 defines a novel monoclonal antibody for prospective isolation of living neonatal and adult astrocytes. *Glia.* 2017;65(6):990-1004.
23. Schmittgen TD, and Livak KJ. Analyzing real-time PCR data by the comparative C(T) method. *Nat Protoc.* 2008;3(6):1101-8.

Supplementary Figures

Suppl. Figure S1: *Strategy for generation and validation of the G-KiRKO mouse*. (**A**) Schematic representation of the generation of the G-KiRKO mouse line. Gfap-Cre:Kiss1r^{loxP} male mice were crossed with homozygous Kiss1r^{loxP} female mice to generate the G-KiRKO line, lacking *Kiss1r* expression in GFAP-positive cells. The combination of primers for genotyping of G-KiRKO mice, i.e., to detect wild-type and loxP-flanked alleles of *Kiss1r* and the recombinant event is shown. (**B**) Screening of genomic recombinant events detected prominent Cre activity in hypothalamic areas, with low to negligible activity in peripheral tissues in G-KiRKO mouse model, except for the testis. Gfap-Cre:Kiss1r^{loxP} mice lacking *Cre* expression served as control. PCR products for detecting recombinant events (750-bp band) are shown. Lower panel in **B** includes an image of primary astrocyte cultures from neonatal G-KiRKO mice (*left side*) and the bar graph showing *Kiss1r* mRNA expression measured by RT-qPCR from hypothalamic astrocytes of G-KiRKO mice (green) and control mice (blue). Whole hypothalamic samples from control mice (white) were used as reference control. *Kiss1r* mRNA levels were normalized using *S11* mRNA. Data are presented as mean ± SEM. Statistical significance was determined by one-way ANOVA followed by Bonferroni's post hoc test: **P<0.01; ****P< 0.0001 vs. corresponding values in control astrocytes. Finally, in **C**, Western blot analyses are shown of pERK, pAKT, total ERK (ERK), total AKT (AKT), GFAP, NeuN (neuronal marker) and β-catenin (used as control; all from the same gel) in primary mouse hypothalamic astrocyte cultures from G-KiRKO mice, treated with Kp-10 (10^{-8} M) at 1- and 10-min. Bar graphs show the effect of Kp-10 over the ratio pERK/ERK and pAKT/AKT, respectively, and GFAP expression. Data are presented as mean ± SEM. Statistical significance was determined by 2-way ANOVA followed by Bonferroni's post hoc test: *P<0.05 corresponding values in astrocyte control treated with Kp-10 vs. vehicle.

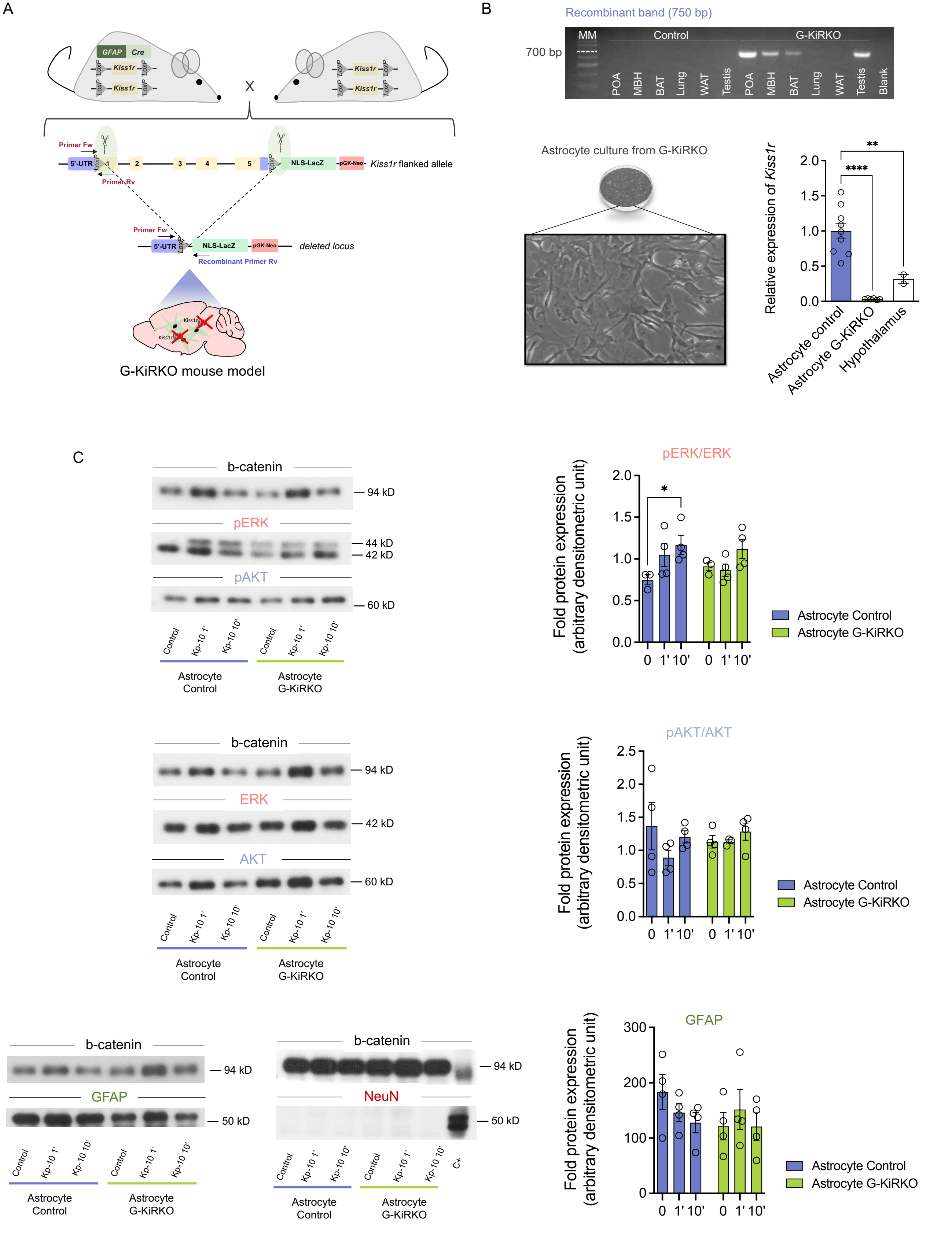

Suppl. Figure S1

Suppl. Figure S2: *Analysis of GFAP-mediated recombination in vivo in the G-KiRKO mouse.* To assess the capacity of our G-KiRKO mouse to drive Cre-mediated ablation of *Kiss1r* in astrocytes, a reporter mouse was generated using the same Gfap-Cre mouse line, which expresses YFP (denoted by GFP-immunoreactivity) following Cre-induced recombination. Immunohistochemical analyses for detection of GFP together with two different astrocyte markers (GFAP and S100β) was conducted in adult female mice at diestrus (N=4). In panel **A**, representative images are shown, at two different magnifications, identifying GFAP- (cyan), S100β- (magenta) and GFP-labelled (green) cells in sections of the ARC. Merge image of all signals documents the co-expression of GFP with the astrocyte markers, GFAP/ S100β, in triple-positive cells. Amplification of discrete areas –dotted square- from each signal are presented underneath, together with a merged image, in which six GFAP/S100β-positive cells co-expressing GFP are shown (white arrows). Scale bar = 100 µm. Similar analyses were conducted using NeuN as neuronal marker. In panel **B**, representative images are presented of NeuN- (magenta) and GFP-labelled (green) cells in the ARC. Merge of these signals denote occasional co-expression in double-positive cells. Amplification of discrete areas –dotted square- and a merge image are shown underneath, in which double positive cells are denoted by white arrows. Scale bar = 100 µm. Quantification of % of triple-positive GFAP/S100β/GFP cells (denoting astrocytes with effective recombination) is shown in **C**, while the % of double-positive NeuN/GFP cells is shown in **D**. Data are presented as mean ± SEM.

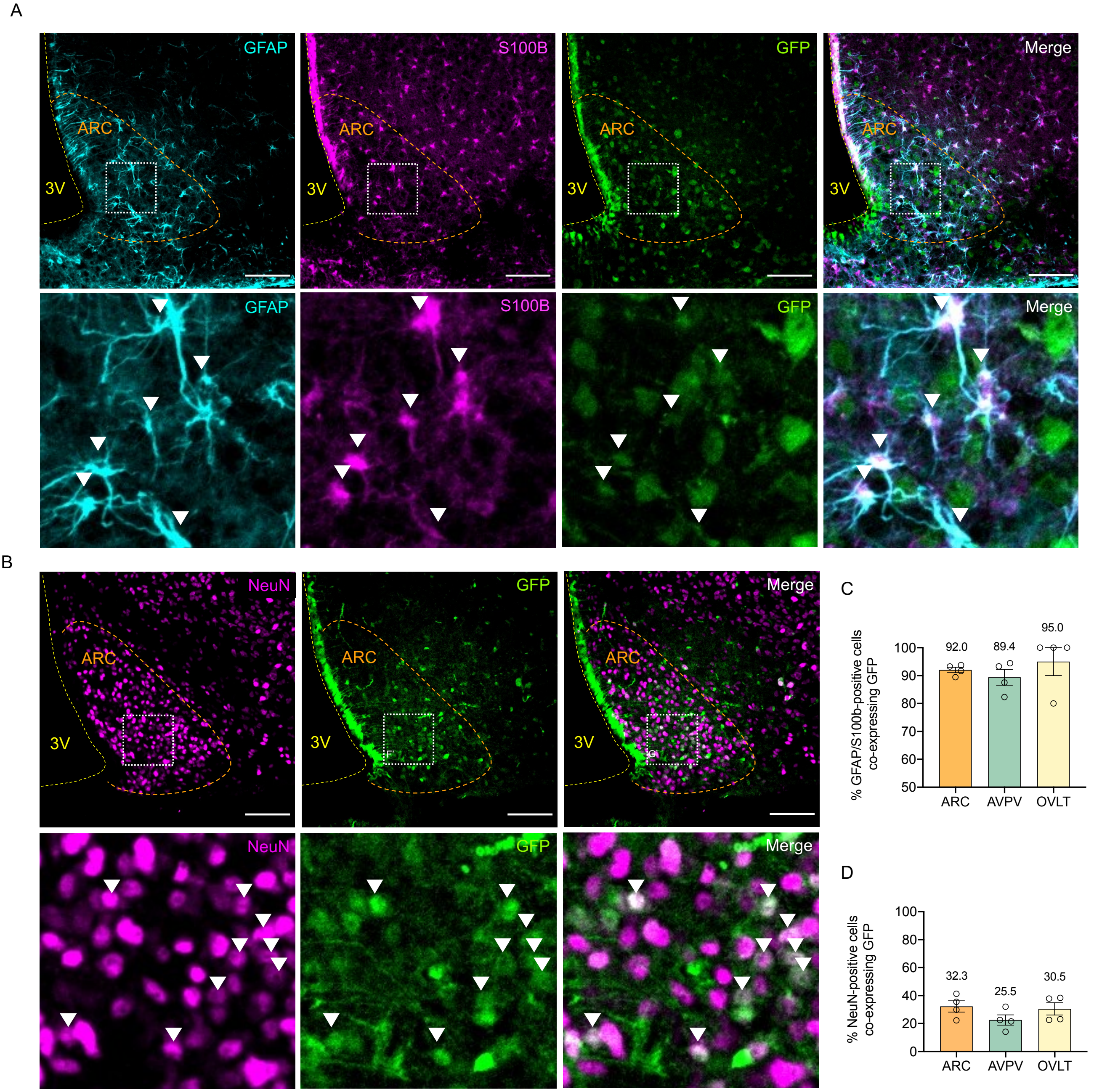

Suppl. Figure S2

Suppl. Figure S3: *Phenotypic characterization of female G-KiRKO mice*. In the upper panel, evolution of body weight (BW) gain from weaning (PND23) to adulthood in female G-KiRKO mice fed with normal diet (**A**) or HFD (**B**) is presented; group sizes: control (n= 8); G-KiRKO (n= 5); control HFD (n= 19); G-KiRKO HFD (n= 9). In addition, accumulated percentages of female mice displaying first estrus (FE) during the period of 3 weeks post weaning, are shown for mice fed with normal diet (**C**) or HFD (**D**); group sizes were as follows: control (n= 16); G-KiRKO (n= 16); control HFD (n= 18); and G-KiRKO HFD (n= 9). In **E**, estradiol-induced LH surge profiles in G-KiRKO mice are shown. LH secretion as time-course profile before lights off (10.00 am and 8 pm) and after lights off (every 15 min till 8.20 pm until 8.50 pm) in ovariectomized control and G-KiRKO mice subjected to estrogen priming are presented. Group sizes were as follows: control (blue, n= 5) and G-KiRKO (green, n= 3). The bar graph shows the net increment of integral (AUC) LH secretion over the preovulatory surge. Individual LH preovulatory peak profiles from control and G-KiRKO mice are shown in the right side of panel. Finally, in **F**, different fecundity indices of G-KiRKO mice are presented. These include fertility rates, breeding intervals and litter sizes from control and G-KiRKO females crossed with control male mice of proven fertility. Data are presented as mean ± SEM.

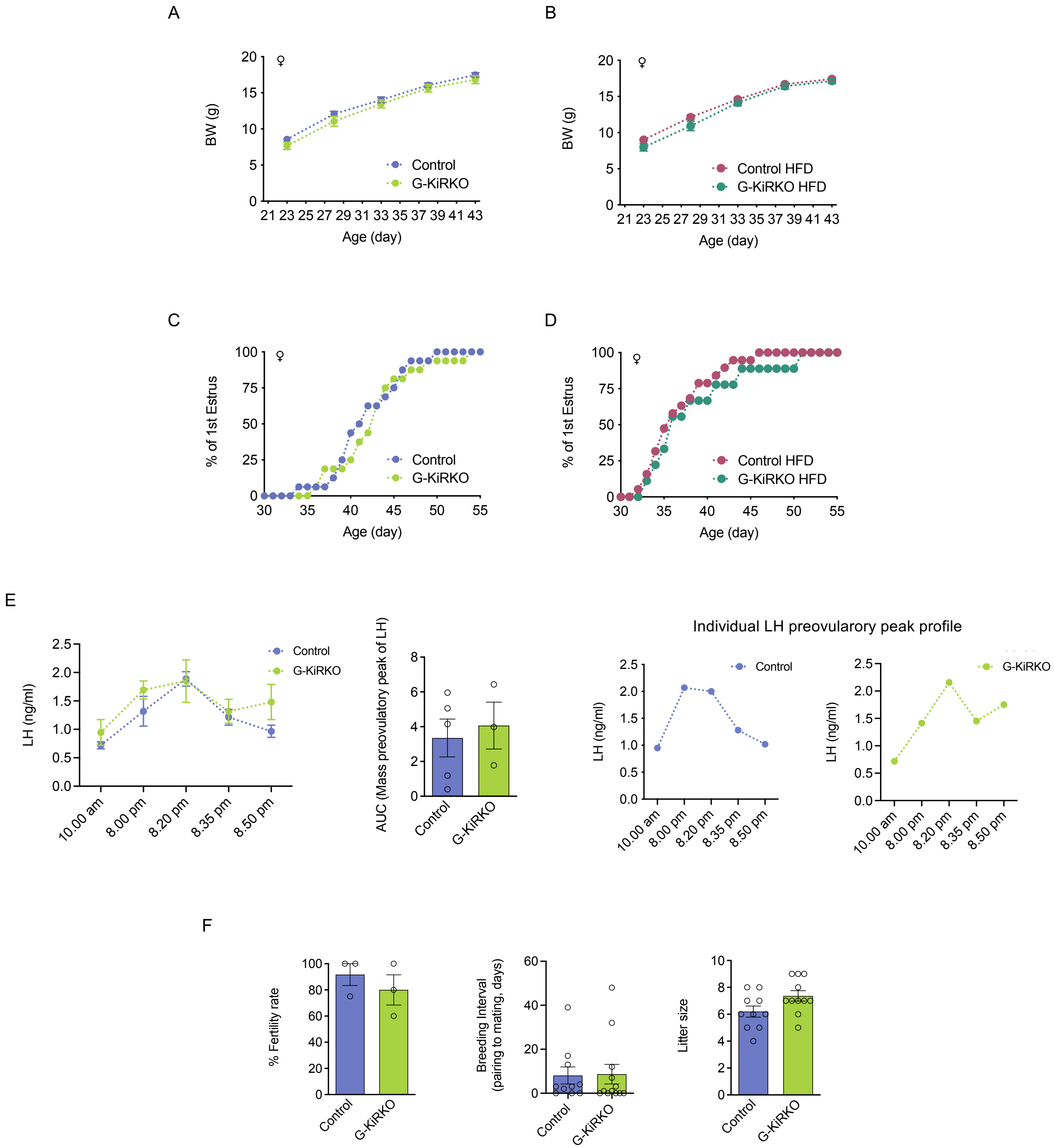

Suppl. Figure S3

Suppl. Figure S4: *Phenotypic characterization of male G-KiRKO mice*. In the upper panels, evolution of body weight (BW) gain from weaning (PND23) to adulthood in male G-KiRKO mice, fed with normal diet (**A**) or HFD (**B**), is shown; group sizes: control (n= 26); G-KiRKO (n= 12); control HFD (n= 21) and G-KiRKO HFD (n= 11). In addition, accumulated percentage of male mice fed with normal diet (**C**) or HFD (**D**) displaying balano-preputial separation (BPS) during the period of 3 weeks post weaning is shown. Group sizes were as follows: control (blue, n= 20); G-KiRKO (n= 12); control HFD (n= 35) and G-KiRKO HFD (n= 17). In **E**, LH secretory profiles are shown as 60-min time-course profile after icv injection of Kp-10 (50 pmol) in male mice fed with normal or HFD. In addition, net increment of integral (AUC) LH secretion over the 60-min period after Kp-10 injection is displayed, for both normal diet and HFD groups; sizes: control (n= 4); G-KiRKO (n= 8); control HFD (n= 9) and G-KiRKO HFD (n= 14). Data are presented as mean ± SEM. Statistical significance was determined by 2-way ANOVA followed by Bonferroni's post hoc test for time-course analyses: *$P<0.05$; $^{\#\#}P<0.01$; $^{\#\#\#}P<0.001$; $^{****/\#\#\#\#}P<0.0001$ vs. corresponding basal (time 0) values; and $^{a}P< 0.05$ G-KiRKO vs. corresponding values in control mice.

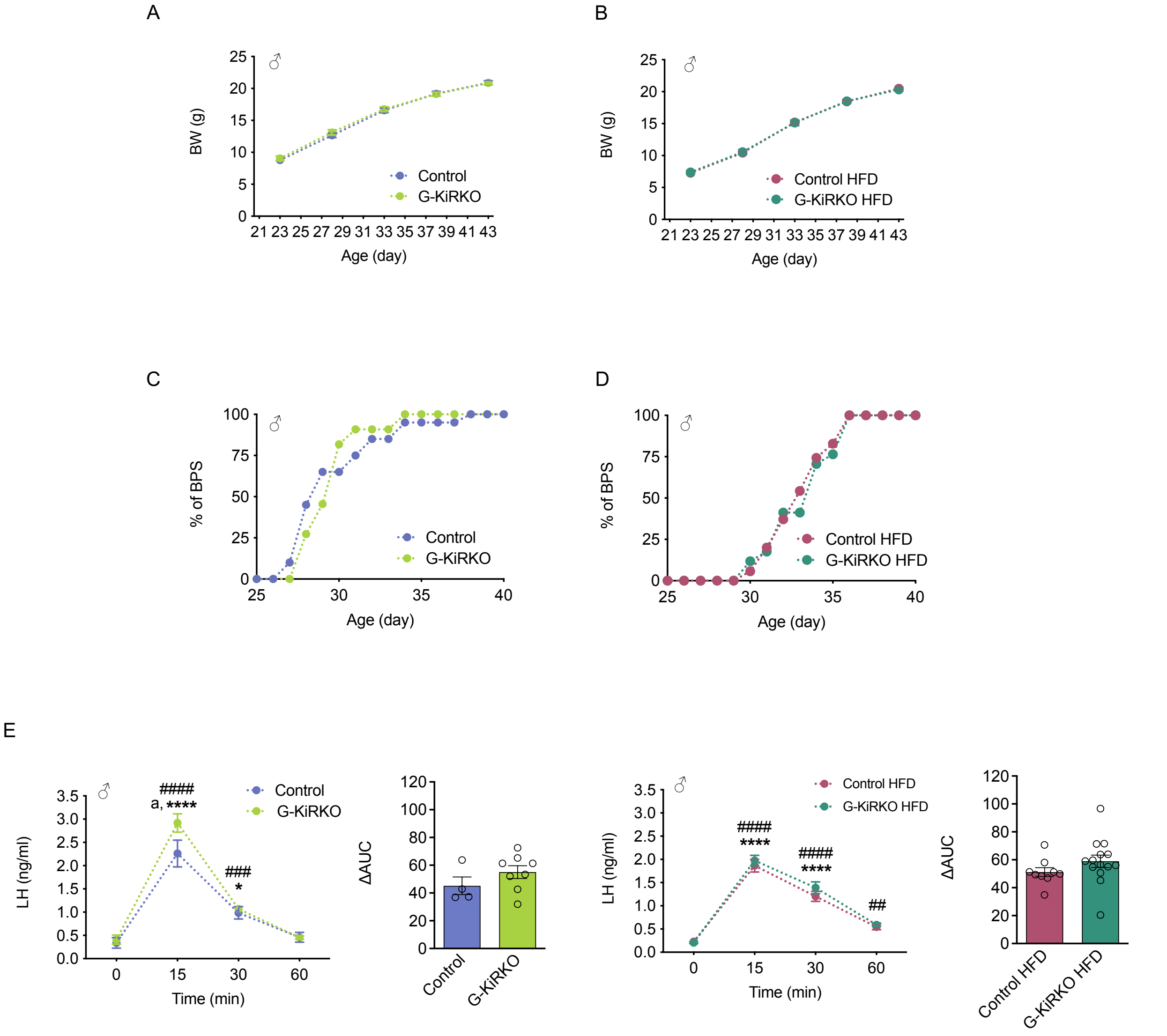

Suppl. Figure S4

Suppl. Figure S5: *Individual LH secretory profiles of female G-KiRKO mice fed control diet or HFD*. (**A**) Three individual representative LH secretion profiles of control and G-KiRKO mice, fed with normal diet, are shown. In addition, in **B**, three individual representative LH secretion profiles of control and G-KiRKO mice fed HFD are presented. The pulsatile parameters of these individuals are included in the quantitative analyses presented in Figure 5, panels G-H.

A

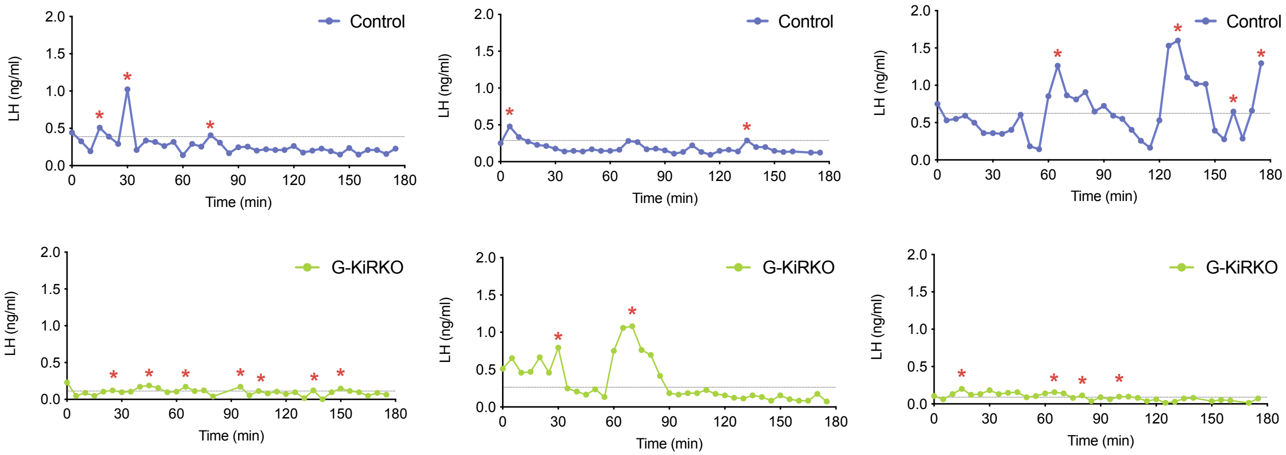

Control
G-KiRKO
LH (ng/ml)
Time (min)

B

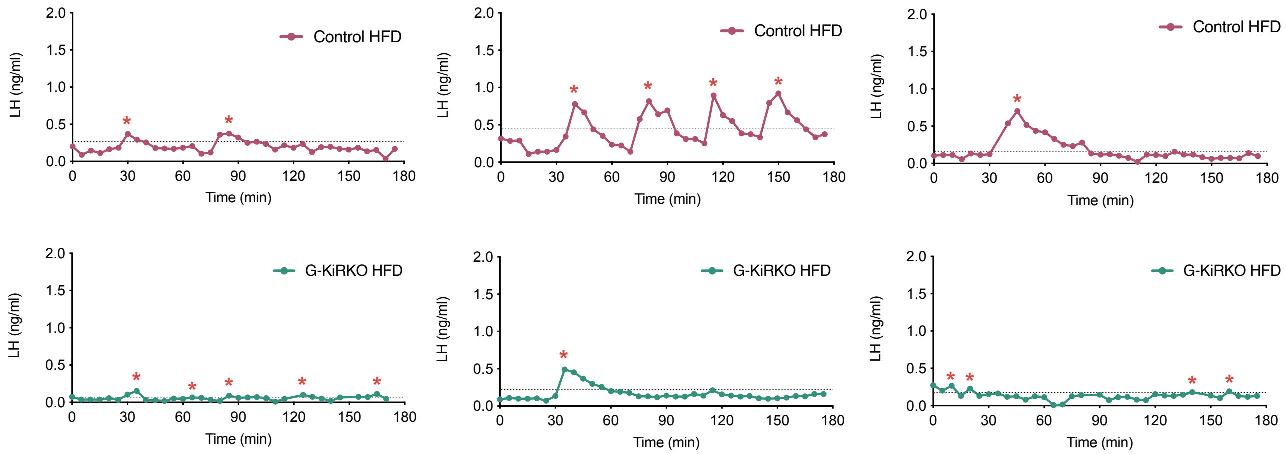

Control HFD
G-KiRKO HFD
LH (ng/ml)
Time (min)

Suppl. Figure S6: *Body weight and composition in G-KiRKO mice*. Metabolic parameters in control and G-KiRKO mice from both sexes, fed normal diet (**A**) or HFD (**B**), are shown. Body weight (BW) gain, and percentages of fat and lean mass are presented, with the following group sizes: female control (n= 7); female G-KiRKO (n= 12); male control (n= 12); male G-KiRKO (n= 8); female control HFD (n= 10-19); female G-KiRKO HFD (n= 6-11); male control HFD (n= 20-33); male G-KiRKO HFD (n= 13-19). Data are presented as mean ± SEM.

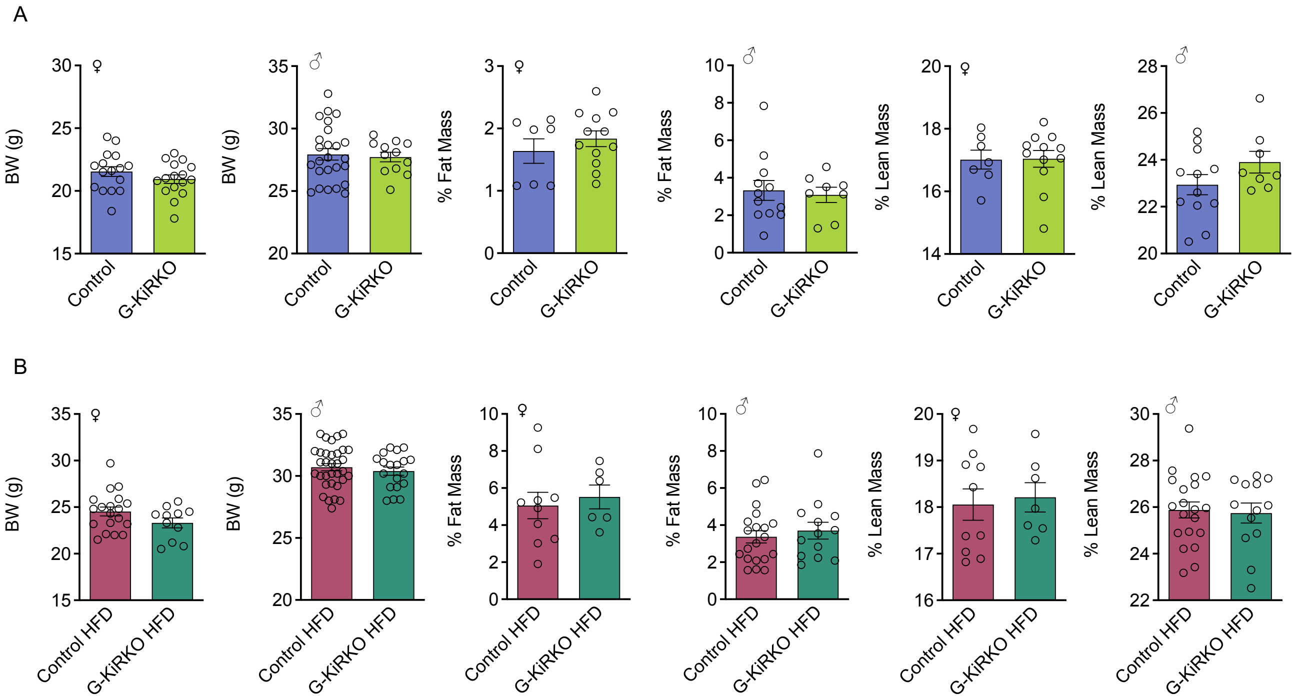

Suppl. Figure S6

Suppl. Figure S7: *Glycemic homeostasis in G-KiRKO mice*. (**A-B**) Results from glucose tolerance tests (GTT), represented as 120-min time-course profile after i.p. injection of a glucose bolus, and net increment of integral (AUC) glucose levels during GTT, are shown from female (**A**) and male (**B**) control and G-KiRKO mice, fed normal diet or HFD. Groups sizes were: female control (n= 8); female G-KiRKO (n= 12); male control (n= 7); male G-KiRKO (n= 10); female control HFD (n= 10); female G-KiRKO HFD (n= 10); male control HFD (n= 11); male G-KiRKO HFD (n= 9). Data are presented as mean ± SEM. Statistical significance was determined by 2-way ANOVA followed by Bonferroni's post hoc test: $^{*/\#}P<0.05$; $^{***}P<0.001$; $^{****/\#\#\#\#}P<0.0001$ vs. corresponding basal (time 0) values; and ^{a} $P<0.05$ G-KiRKO vs. corresponding values in control mice; and by Student's t-test (AUC values): $^{*}P<0.05$ vs. corresponding values in control mice. (**C-D**) Results from insulin tolerance tests (ITT), represented as 120-min time-course profile after i.p. injection of an insulin bolus, and net increment of integral (AUC) glucose levels during ITT, are shown from female (**C**) and male (**D**) control and G-KiRKO mice, fed normal diet or HFD. Groups sizes were: female control (n= 8); female G-KiRKO (n= 12); male control (n= 6); male G-KiRKO (n= 9); female control HFD (n= 10); female G-KiRKO HFD (n= 10); male control HFD (n= 10); male G-KiRKO HFD (n= 10). Data are presented as mean ± SEM. Statistical significance was determined by 2-way ANOVA followed by Bonferroni's post hoc test: $^{**/\#\#}P<0.01$; $^{***/\#\#\#}P<0.001$; $^{****/\#\#\#\#}P<0.0001$ vs. corresponding basal (time 0) values; and ^{a} $P<0.05$ G-KiRKO vs. corresponding values in control mice; and by Student's t-test (AUC values).

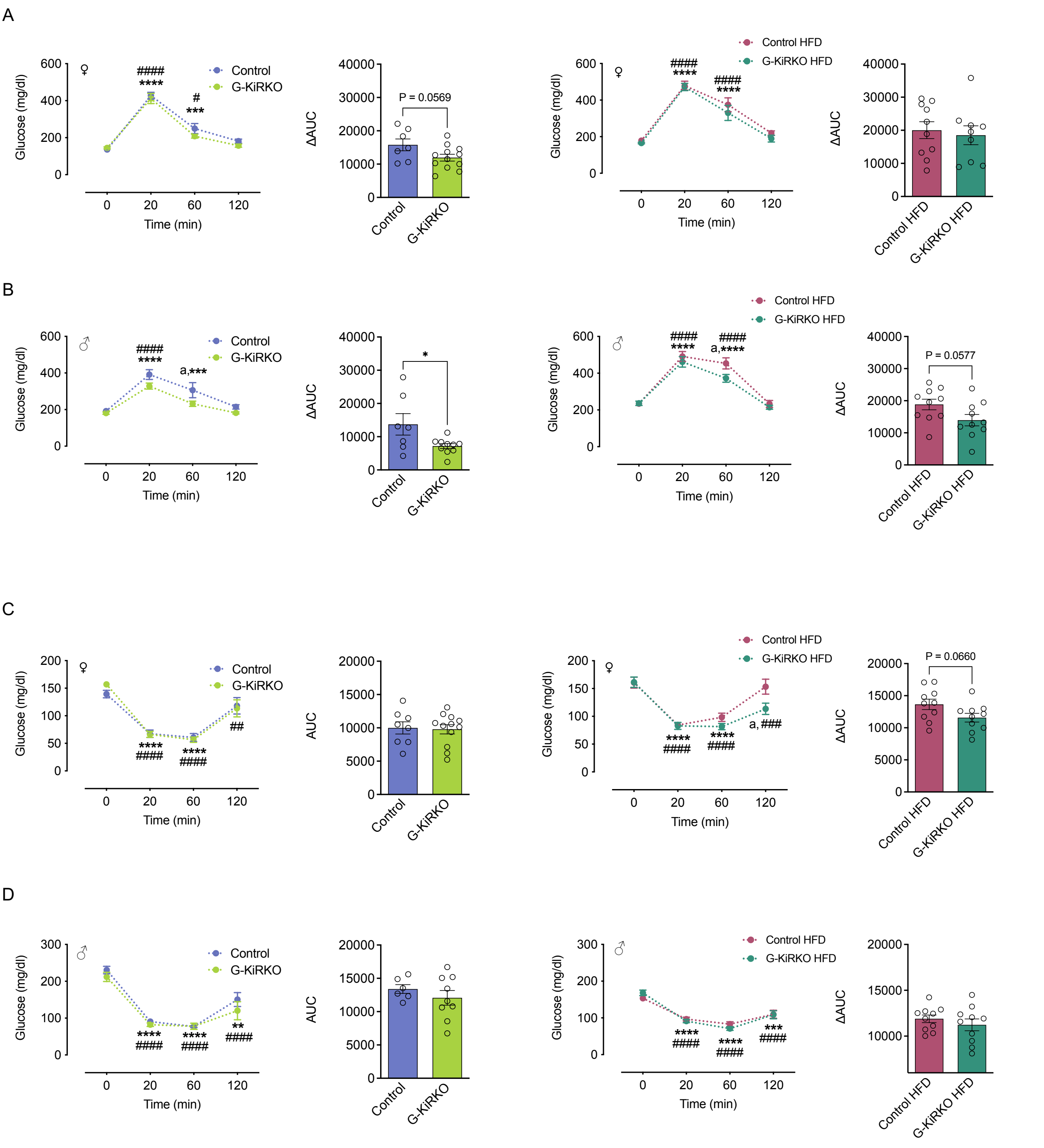

Suppl. Figure S7

Suppl. Figure S8: *GnRH neuron activation and appositions with astrocytes in GKiRKO mice.* We interrogated whether the lack of kisspeptin signaling in astrocytes affects cFos activation in GnRH neurons in response to a bolus of Kp54, as well as the close interactions between GnRH neurons and astrocytes. In (**A**), representative images GnRH- (green) and cFos- (magenta) labelled cells in sections of the OVLT are shown from both control and G-KiRKO mice. Merge images of the two signals document co-expression of cFos in GnRH neurons. Amplification of discrete areas (see cyan-line marking) from control and G-KiRKO mice are also presented, where GnRH neurons co-expressing cFos are indicated with white arrows. In (**B**), representative images of GnRH- (green) and GFAP- (magenta) labelled cells are shown in sections of OVLT for both control and G-KiRKO mice. Merge images document close appositions between GnRH neurons and GFAP-positive cells with the OVLT. Amplification of discrete areas (cyan-line marking) from control and G-KiRKO mice are shown also underneath for each genotype, in which close appositions between GnRH neurons and GFAP-positive cells are indicated with white arrows. In (**C**), LH levels 60 minutes after ip Kp-54 administration, denoting effective activation of the gonadotropic axis, are presented; basal LH levels before Kp-54 injection are denoted by the dotted red line. In (**D**), the percentage of GnRH neurons co-expressing cFos in the preoptic area (POA) after Kp-54 administration is shown for control and GKiRKO mice. In addition, the percentage of GnRH neurons in close apposition with GFAP-positive cells is presented, either by contacts at the level of the soma (**E**) or GnRH processes at POA (**F**). Data are presented as mean ± SEM. Experiments were conducted in diestrus control and G-KiRKO adult female mice (n=5/group). Statistical significance was determined by Student's t-test: $^*P<0.05$ vs. corresponding to control values.

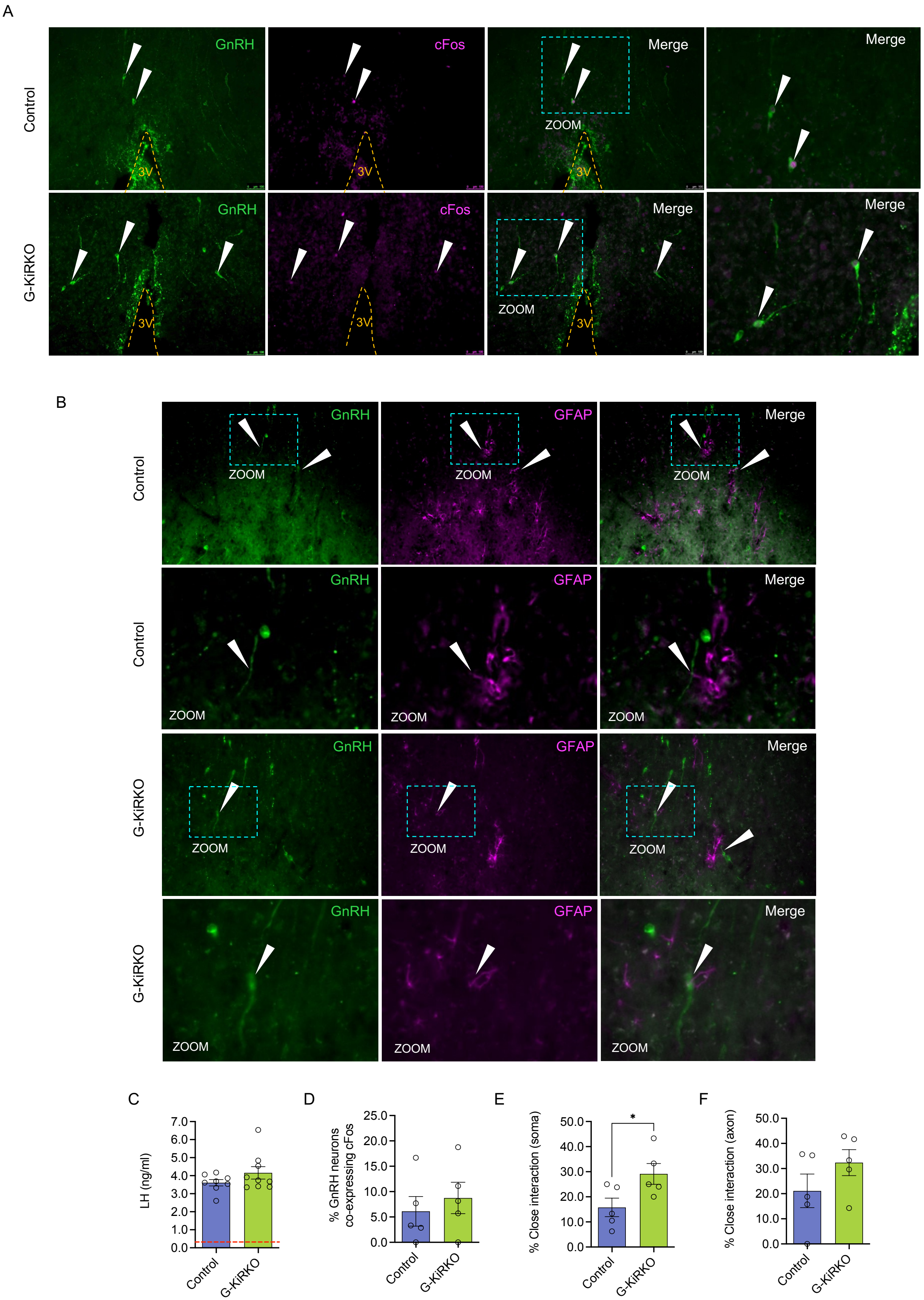

Suppl. Figure S8

Suppl. Table S1: *Comparative phenotypes of GFAP-specific Kiss1r KO (G-KiRKO) mice vs. other mouse models of conditional or global ablation of Kiss1r*. Reproductive and metabolic phenotypes, as well as hormonal features of **G-KiRKO** mice are compared with those of models of (*a*) global ablation of Kiss1r (**global KO**), (*b*) conditional ablation of Kiss1r in GnRH neurons (**GnRH-specific KO**); and (*c*) conditional ablation of Kiss1r in POMC neurons (**POMC-specific KO**). Source papers for each parameter are quoted numerically, as from the reference list below.

		G-KiRKO	Global KO	GnRH-specific KO	POMC-specific KO
**Puberty onset**		Normal	Absent[1]	Absent[2]<br>Delayed[5]	Normal[3]
**Fertility**		Preserved	Absent[1,4]	Absent[2,5]	Preserved[3]
**LH secretion**	**Responses to Kp-10**	Augmented	Absent[4,6]	Absent[5]	Conserved[3]
	**LH pulsatility**	Normal mean LH	↓ mean LH[4] /<br>Normal mean LH[1,6]	↓ mean LH[5]	Normal mean LH[3]
		↓ basal LH	↓ basal LH[7]	NT	NT
		Normal # LH peaks<br>↓ LH peak mass after HFD	Fewer but detectable LH peaks[7]	NT	NT
	**Pre-ovulatory surge**	Preserved	Absent[8,9]	Likely absent[2]<br>(no ovulation)	Likely preserved[3]<br>(Preserved fertility)
**Metabolic parameters**		Normal BW	↑ BW (females)[10]<br>↓ BW (males)[11]	Normal BW[5]	Normal BW[3]
		Normal BC	↑ adiposity[10,11]	NT	NT
		Improved glucose tolerance	Worsen glucose tolerance (female)[10]	NT	NT
		Normal insulin sensitivity	Normal insulin sensitivity[11]	NT	NT

HFD, high fat diet; **BW**, body weight; **BC**, body composition; **NT**, not tested

Note: We refer to "mean LH" as the average of LH levels in single-point determinations, while "basal LH" refers to baseline levels of LH in pulsatile analyses from serial sampling studies.

References

1. Seminara, S.B., *et al.* The GPR54 gene as a regulator of puberty. *N Engl J Med* **349**, 1614-1627 (2003).
2. Kirilov, M., *et al.* Dependence of fertility on kisspeptin-Gpr54 signaling at the GnRH neuron. *Nat Commun* **4**, 2492 (2013).
3. Manfredi-Lozano, M., *et al.* Defining a novel leptin-melanocortin-kisspeptin pathway involved in the metabolic control of puberty. *Mol Metab* **5**, 844-857 (2016).
4. Garcia-Galiano, D., *et al.* Kisspeptin signaling is indispensable for neurokinin B, but not glutamate, stimulation of gonadotropin secretion in mice. *Endocrinology* **153**, 316-328 (2012).
5. Novaira, H.J., *et al.* Disrupted kisspeptin signaling in GnRH neurons leads to hypogonadotrophic hypogonadism. *Mol Endocrinol* **28**, 225-238 (2014).
6. Lapatto, R., *et al.* Kiss1-/- mice exhibit more variable hypogonadism than Gpr54-/- mice. *Endocrinology* **148**, 4927-4936 (2007).
7. Steyn, F.J., *et al.* Development of a methodology for and assessment of pulsatile luteinizing hormone secretion in juvenile and adult male mice. *Endocrinology* **154**, 4939-4945 (2013).
8. Clarkson, J., d'Anglemont de Tassigny, X., Moreno, A.S., Colledge, W.H. & Herbison, A.E. Kisspeptin-GPR54 signaling is essential for preovulatory gonadotropin-releasing hormone neuron activation and the luteinizing hormone surge. *J Neurosci* **28**, 8691-8697 (2008).
9. Dror, T., Franks, J. & Kauffman, A.S. Analysis of multiple positive feedback paradigms demonstrates a complete absence of LH surges and GnRH activation in mice lacking kisspeptin signaling. *Biol Reprod* **88**, 146 (2013).
10. Tolson, K.P., *et al.* Impaired kisspeptin signaling decreases metabolism and promotes glucose intolerance and obesity. *J Clin Invest* **124**, 3075-3079 (2014).
11. Velasco, I., *et al.* Gonadal hormone-dependent vs. -independent effects of kisspeptin signaling in the control of body weight and metabolic homeostasis. *Metabolism* **98**, 84-94 (2019).

Suppl. Table S2: *Primers and conditions used for qPCR analyses.*

Targets	Sequence (5'-3')	Annealing Temp (º C)	Amplicon Size (bp)
Cdk2 (NM_183417.3)<br>Forward<br>Reverse	GAAGATGGACGGAGCTTGTT<br>AGGTGGGGCACTGGTTTAG	60	119
Cox-1 (NM_008969.4)<br>Forward<br>Reverse	GATTGTACTCGCACGGGCTAC<br>GGATAAGGTTGGACCGCACT	60	202
Cox-2 (NM_011198.4)<br>Forward<br>Reverse	CAGCCAGGCAGCAAATCC<br>ACATTCCCCACGGTTTTGAC	60	55
cPges (NM_019766.4)<br>Forward<br>Reverse	GGAAAGACTGGGAGGATGACTC<br>TCATCTGCTCCATCTACTTCTGG	60	121
Gfap (NM_010277.3)<br>Forward<br>Reverse	CACCTACAGGAAATTGCTGGAGG<br>CCACGATGTTCCTCTTGAGGTG	60	137
Hsd3b1 (NM_008293.4)<br>Forward<br>Reverse	AGAACTGCAGGAGGTCAGAGCT<br>GGCATCCAGAATGTCTCCTTCC	60	118
Kiss1 (NM_178260.3)<br>Forward<br>Reverse	GCTGCTGCTTCTCCTCTGTG<br>TCTGCATACCGCGATTCCTT	60	129
Kiss1r (NM_001359010.1)<br>Forward<br>Reverse	CGGAAACTCATTGGTCA<br>TGTGGCTTGCACCGAGA	65	214
Ki67 (NM_001081117.2)<br>Forward<br>Reverse	ACAGACTTGCTCTGGCCTACC<br>CTTCCTCTTGGTTGGCGTTT	60	196
mPges (NM_022415.3)<br>Forward<br>Reverse	GTGGTTTCAGCAGGGTGTCACT<br>GTCTTGAGTCCAGATTTGCAGC	60	129
mPges (NM_022415.3)<br>Forward<br>Reverse	GGTCATCAAGATGTACGCGG<br>ATCCTCGGGGTTGGCAAAAG	60	79
Nanog (NM_028016.3)<br>Forward<br>Reverse	CCTCCAGCAGATGCAAGAACT<br>ATGCTGGGATACTCCACTGGT	60	171

Ncam1 (NM_001113204.1)			
Forward	GGTTCCGAGATGGTCAGTTGCT	60	158
Reverse	CAAGGACTCCTGTCCAATACGG		
Pges2 (NM_133783.1)			
Forward	GGTAGACCTCTATGAAGCAGCC	60	121
Reverse	CATCACTCGCAGCACACCATAC		
P450arom (NM_001348171.1)			
Forward	CGAAGCAGCAATCCTGAAGGAG	60	134
Reverse	CCAAGTCCACAACAGGCTGGTA		
P450scc (NM_019779.4)			
Forward	TGCTCAACCTGCCTCCAGACTT	60	150
Reverse	ACTGGCTGAAGTCTCGCTTCTG		
Rpl19 (NM_009078.2)			
Forward	GAAATCGCCAATGCCAACTC	61	290
Reverse	ACCTTCAGGTACAGGCTGTG		
Sox-2 (NM_011443.4)			
Forward	ACAGCATGATGCAGGAGCAG	60	175
Reverse	TGCGAGTAGGACATGCTGTAG		
Star (NM_011485.5)			
Forward	GTGCTTCATCCACTGGCTGGAA	60	113
Reverse	GTCTGCGATAGGACCTGGTTGA		
SynCam1 (NM_207675.2)			
Forward	ACTTCTGCCAGCTCTACACGGA	60	113
Reverse	CCCTTCAACTGCCGTGTCTTTC		
S11 (NM_013725.4)			
Forward	CATTCAGACGGAGCGTGCTTAC	58	240
Reverse	TGCATCTTCATCTTCGTCAC		
Tspo (NM_009775.4)	GAGCCTACTTTGTACGTGGCGA		
Forward	GCTCTTTCCAGACTATGTAGGAG	60	147
Reverse			
Vimentin (NM_011701.4)			
Forward	CGGAAAGTGGAATCCTTGCAGG	58	138
Reverse	AGCAGTGAGGTCAGGCTTGGAA		

Suppl. Table S3: *Antibodies used for immunohistochemical (IHC) analyses*. Comprehensive list of antibodies used for IHC, including reference/source, host species and dilutions. Antibodies used for triple immunofluorescence detection of kisspeptin, GnRH and GFAP are indicated with an asterisk. Antibodies used for double immunofluorescence detection of cFos and GnRH are indicated with two asterisks. Antibodies used together with RNAscope are marked with three asterisks.

Antibody	Reference	Host species	Dilution
Anti-GFAP	Cell Signaling 3670S	mouse	1/500
Anti-GFP	Abcam ab13970	chicken	1/2000
Anti-S100β	Abcam ab41548	rabbit	1/1000
Anti-mouse Alexa 555	Thermo Fisher A-31570	donkey	1/500
Anti-chicken Alexa 488	Thermo Fisher A-11039	goat	1/500
Biotin anti-rabbit	Jackson Inmuno-Research 711-066-152	donkey	1/500
Anti-rabbit Alexa 647	Thermo Fisher A-21244	goat	1/500
Anti-Kisspeptin	AC-566 (Caraty)	rabbit	1/10000
Anti-GFAP*	Millipore-Sigma AB5541	chicken	1/2000
Anti-kisspeptin*	Millipore-Sigma AB9754	rabbit	1/1000
Anti-GnRH*	Millipore-Sigma SAB1403875	mouse	1/1000
Anti-mouse Alexa 488*	Thermo Fisher A-21202	donkey	1/500
Anti-rabbit Alexa 647*	Thermo Fisher A-31573	donkey	1/500
Anti-chicken Alexa 594*	Thermo Fisher A-11042	goat	1/500

Anti-GnRH**	ImmunoStar<br>20075	rabbit	1/1000
Anti-cFos**	Synaptic System<br>226 308	guinea pig	1/500
Anti-rabbit Alexa 568**	Abcam<br>ab175480	donkey	1/500
Anti-guinea pig Alexa 488**	Jackson<br>ImmunoResearch<br>706-545-148	donkey	1/500
Anti-GFAP***	DAKO<br>Z0334	rabbit	1/100
anti-S100β***	DAKO<br>IR504	rabbit	1/100
Anti-rabbit Alexa 488***	Thermo Fisher<br>A-21206	donkey	1/500
Anti-rabbit Alexa 647***	Thermo Fisher<br>A-31573	donkey	1/500

Suppl. Table S4: *Antibodies used for Western blot analyses*. Comprehensive list of antibodies used for Western blots, incl. reference/source, host species and dilutions.

Antibody	Reference	Host species	Dilution
GFAP	Cell Signaling 3670S	mouse	1/1000
NeuN	Abcam ab177487	rabbit	1/5000
Vimentin (D21H3)	Cell Signaling 5741	rabbit	1/1000
ß -catenin	Sigma-Aldrich C2206	rabbit	1/4000
ERK	Santa Cruz Biotechnology sc-154	rabbit	1/1000
Phospho-p44/42 MAPK	Cell Signaling 4370	rabbit	1/1000
AKT	Cell Signaling 9272	rabbit	1/1000
Phospho-AKT	Cell Signaling 9271	rabbit	1/1000
Actin	Sigma-Aldrich A5060	rabbit	1/5000
Anti-mouse	Abcam ab6789	goat	1/5000 - 1/10000
Anti-rabbit	Abcam ab6721	goat	1/2000 - 1/5000
