# Supplementary material for: Kisspeptin signaling in astrocytes modulates the reproductive axis
Source: J Clin Invest. 2024 Jun 11;134(15):e172908. doi: 10.1172/JCI172908 (PMC11291270; doi:10.1172/JCI172908)

Figure 2A

Left panel

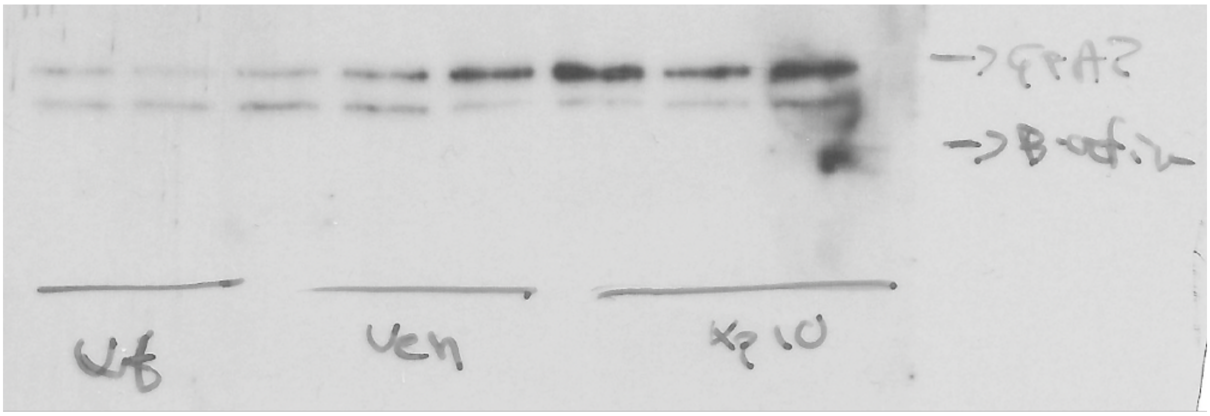

Right panel

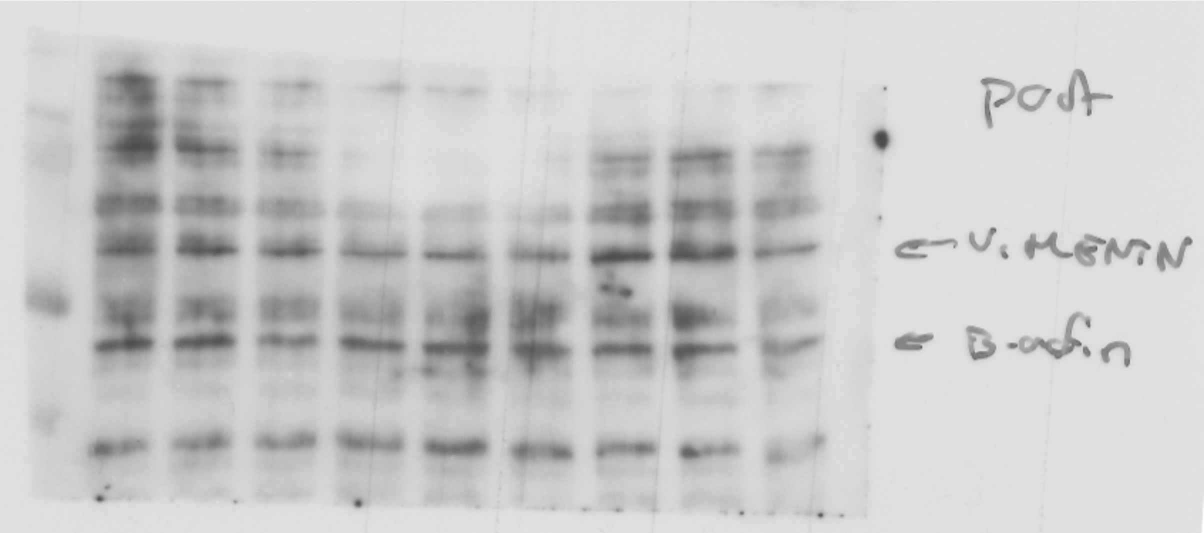

Figure 2C

pERK 1’

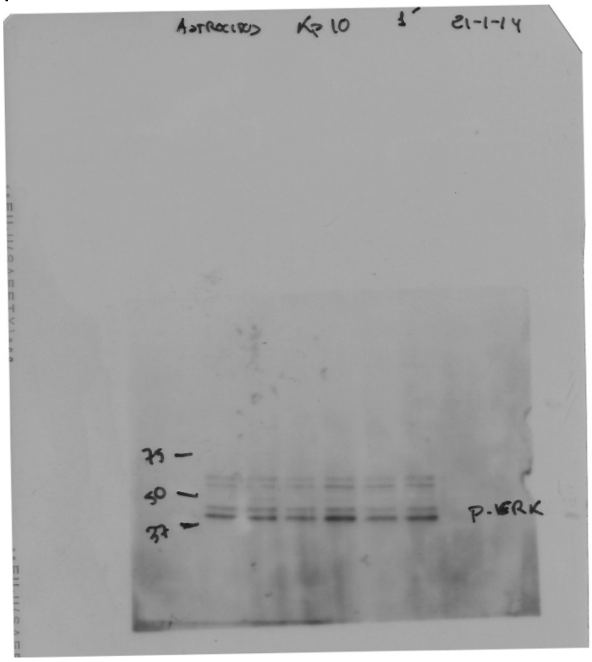

pERK 10’ & 30’

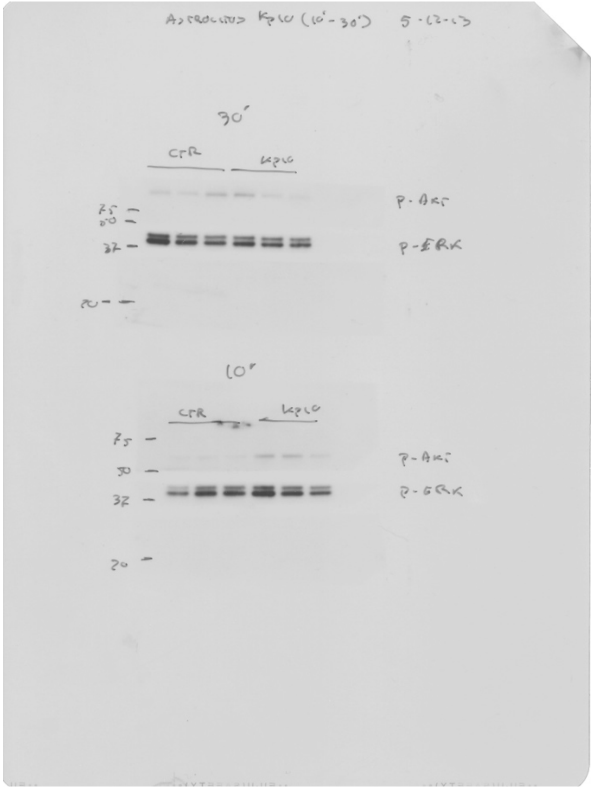

pAKT 1’

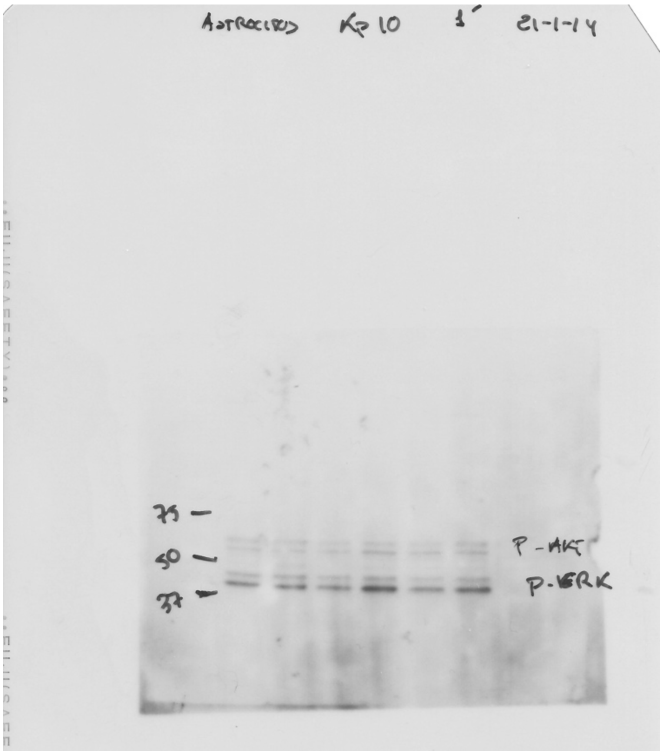

pAKT 10’ & 30’

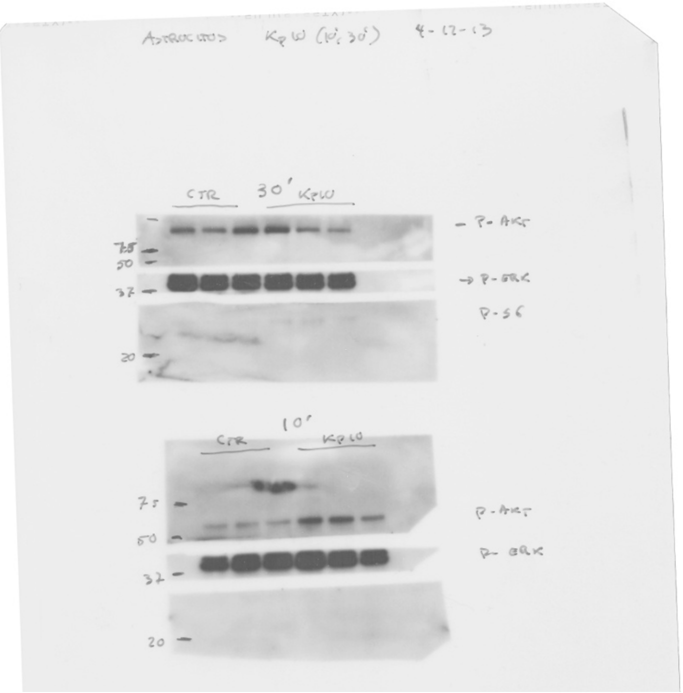

Figure 2D

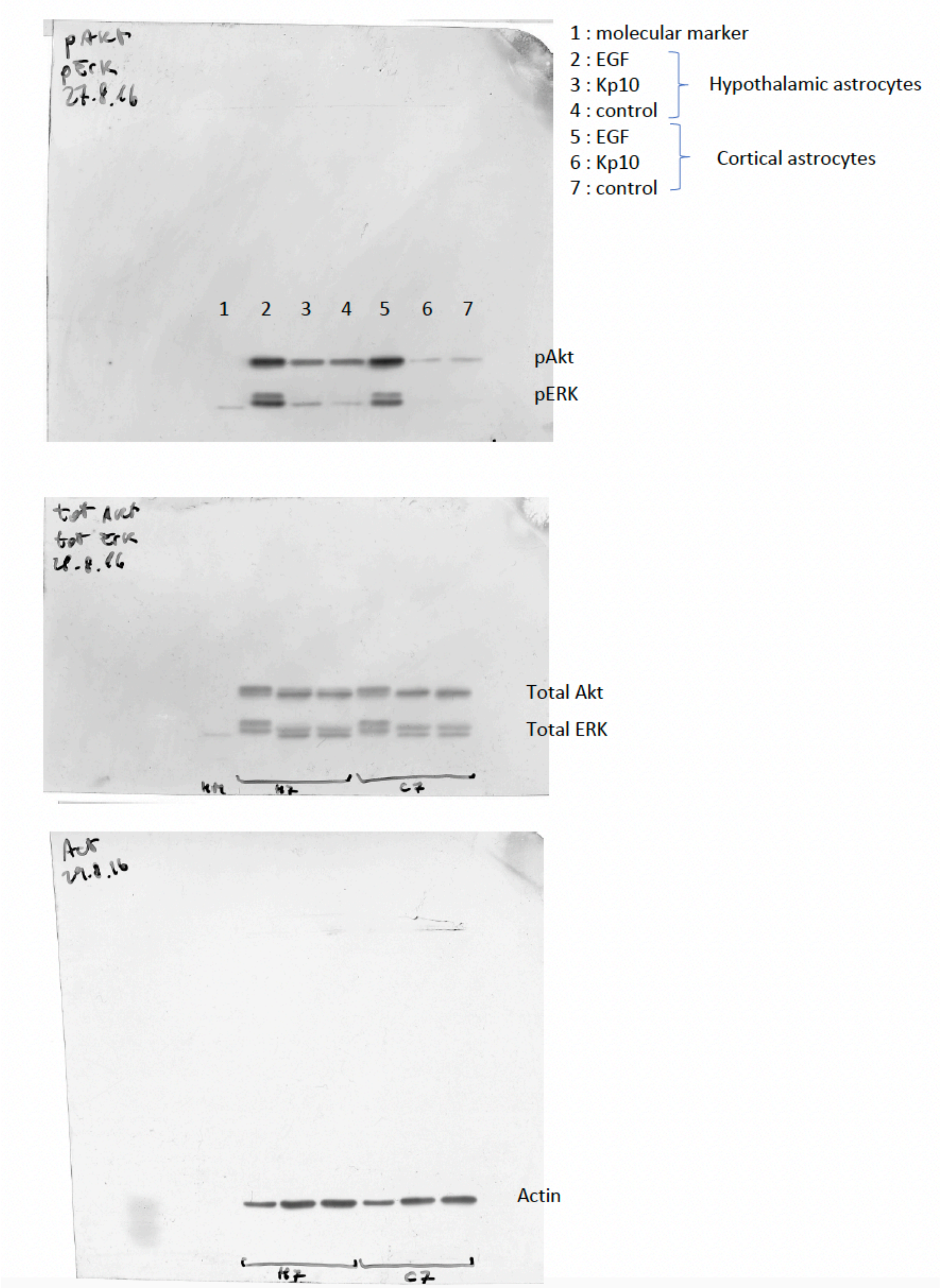
1 : molecular marker
2 : EGF
3 : Kp10
4 : control
Hypothalamic astrocytes
5 : EGF
6 : Kp10
7 : control
Cortical astrocytes
1 2 3 4 5 6 7
pAkt
pERK
Total Akt
Total ERK
Actin

Suppl. Figure 1C

b-catenin, pERK & ERK geles

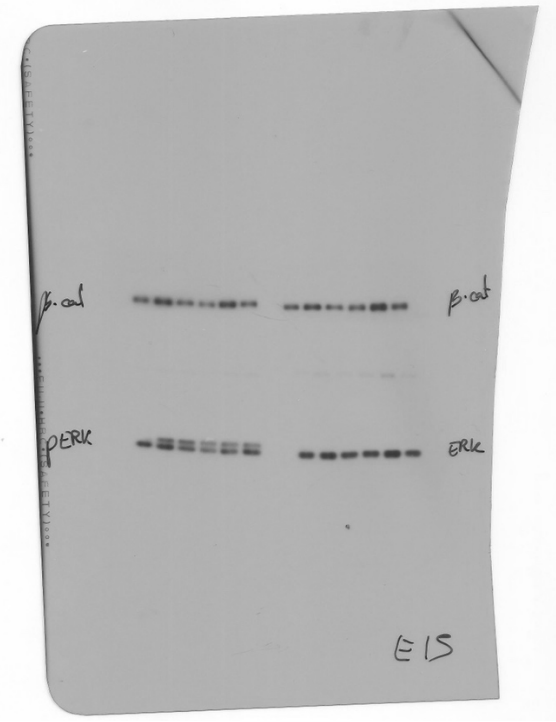

pAKT & AKT geles

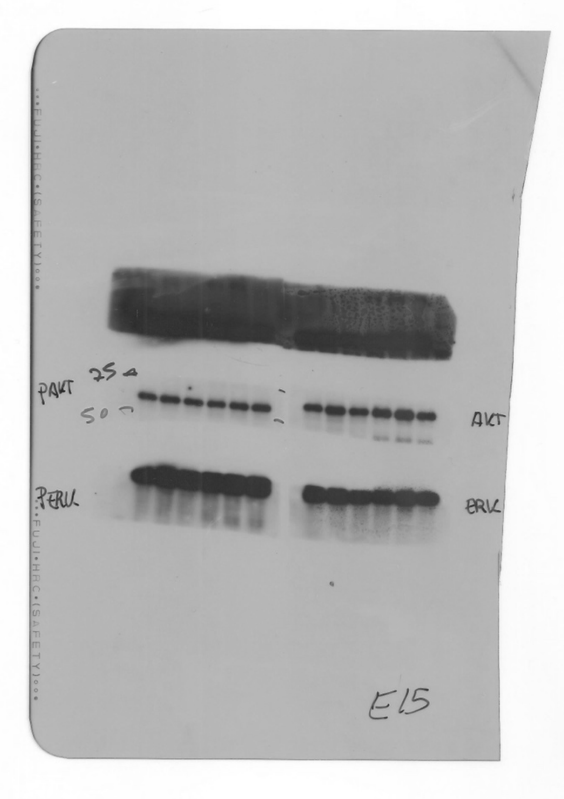

b-catenin & GFAP

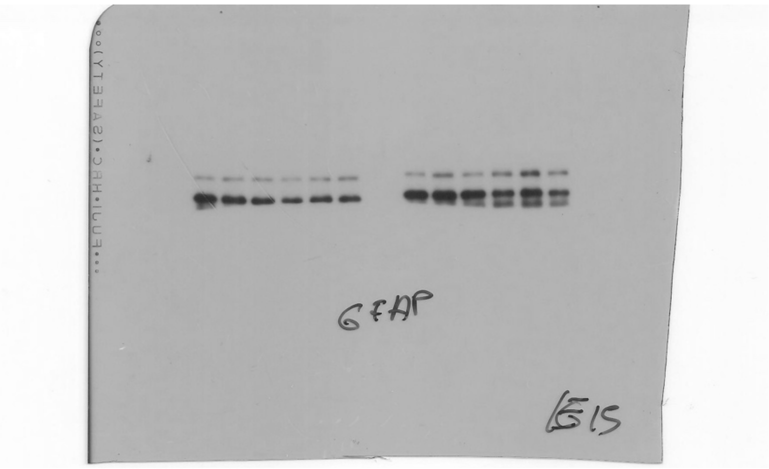

b-catenin & NeuN

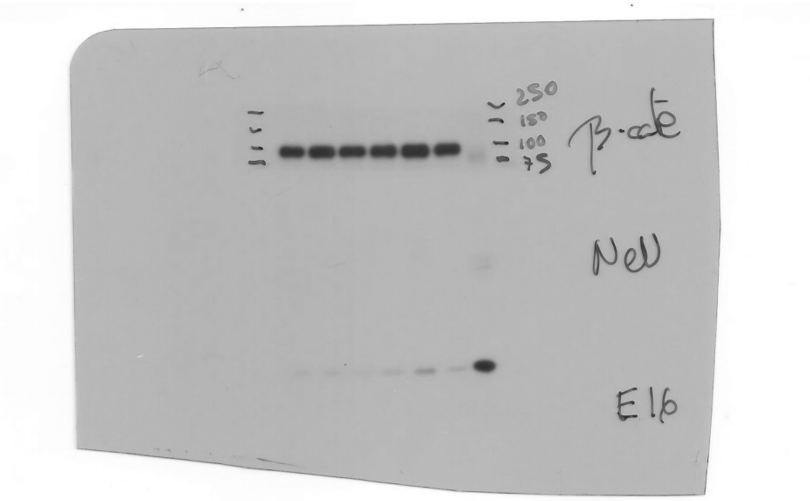

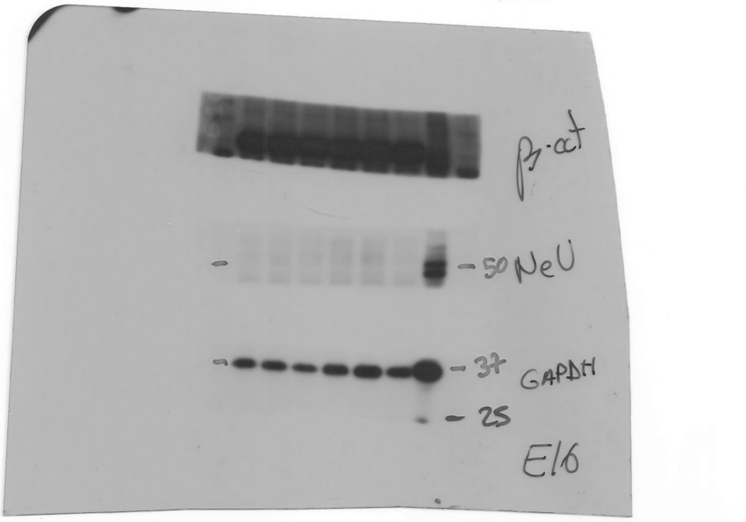

Supplement: Unedited blot and gel images [file jci-134-172908-s171.pdf]
